# Human Monoclonal Antibodies to a Novel Cluster of Conformational Epitopes on HCV E2 with Resistance to Neutralization Escape in a Genotype 2a Isolate

**DOI:** 10.1371/journal.ppat.1002653

**Published:** 2012-04-12

**Authors:** Zhen-yong Keck, Jinming Xia, Yong Wang, Wenyan Wang, Thomas Krey, Jannick Prentoe, Thomas Carlsen, Angela Ying-Jian Li, Arvind H. Patel, Stanley M. Lemon, Jens Bukh, Felix A. Rey, Steven K. H. Foung

**Affiliations:** 1 Department of Pathology, Stanford University School of Medicine, Stanford, California, United States of America; 2 Institut Pasteur, CNRS URA3015, Unite de Virologie Structurale, Paris, France; 3 Copenhagen Hepatitis C Program (CO-HEP), Department of Infectious Diseases and Clinical Research Centre, Copenhagen University Hospital, Hvidovre, Denmark; 4 Department of International Health, Immunology and Microbiology, Faculty of Health Sciences, University of Copenhagen, Copenhagen, Denmark; 5 MRC – University of Glasgow Centre for Virus Research, Glasgow, United Kingdom; 6 Lineberger Comprehensive Cancer Center and the Division of Infectious Diseases, Department of Medicine, The University of North Carolina at Chapel Hill, Chapel Hill, North Carolina, United States of America; Washington University School of Medicine, United States of America

## Abstract

The majority of broadly neutralizing antibodies to hepatitis C virus (HCV) are against conformational epitopes on the E2 glycoprotein. Many of them recognize overlapping epitopes in a cluster, designated as antigenic domain B, that contains residues G530 and D535. To gain information on other regions that will be relevant for vaccine design, we employed yeast surface display of antibodies that bound to genotype 1a H77C E2 mutant proteins containing a substitution either at Y632A (to avoid selecting non-neutralizing antibodies) or D535A. A panel of nine human monoclonal antibodies (HMAbs) was isolated and designated as HC-84-related antibodies. Each HMAb neutralized cell culture infectious HCV (HCVcc) with genotypes 1–6 envelope proteins with varying profiles, and each inhibited E2 binding to the viral receptor CD81. Five of these antibodies neutralized representative genotypes 1–6 HCVcc. Epitope mapping identified a cluster of overlapping epitopes that included nine contact residues in two E2 regions encompassing aa418–446 and aa611–616. Effect on virus entry was measured using H77C HCV retroviral pseudoparticles, HCVpp, bearing an alanine substitution at each of the contact residues. Seven of ten mutant HCVpp showed over 90% reduction compared to wild-type HCVpp and two others showed approximately 80% reduction. Interestingly, four of these antibodies bound to a linear E2 synthetic peptide encompassing aa434–446. This region on E2 has been proposed to elicit non-neutralizing antibodies in humans that interfere with neutralizing antibodies directed at an adjacent E2 region from aa410–425. The isolation of four HC-84 HMAbs binding to the peptide, aa434–446, proves that some antibodies to this region are to highly conserved epitopes mediating broad virus neutralization. Indeed, when HCVcc were passaged in the presence of each of these antibodies, virus escape was not observed. Thus, the cluster of HC-84 epitopes, designated as antigenic domain D, is relevant for vaccine design for this highly diverse virus.

## Author Summary

Hepatitis C virus (HCV) is a highly diverse virus and a significant challenge for vaccine development is to identify protective epitopes conserved in the majority of viral genotypes and subtypes. This problem is compounded by the fact that the envelope E1E2 proteins, the targets for neutralizing antibody response, are two of the most variable proteins of the virus. Modified E2 antigens were constructed that are not bound by antibodies to previously recognized clusters of highly immunogenic epitopes on E2. Their employment as screening antigens has led to the isolation of a novel panel of human monoclonal antibodies to HCV E2. Functional and biochemical studies revealed that these antibodies bind and neutralize HCV of different genotypes and subtypes. Several of these antibodies neutralized cell culture infectious HCV with genotypes 1–6 envelope proteins. Furthermore, when virus was passaged in culture in the presence of each of these antibodies, virus escape was not observed. Thus, these epitopes are relevant in vaccine design for this virus.

## Introduction

Hepatitis C virus (HCV) infection continues to be a major health problem worldwide, and is associated with cirrhosis, liver failure and hepatocellular carcinoma. Nearly 170 million people are chronically infected with HCV and the annual increase in the global burden is estimated at two million new infections [1], [2]. The recent advances in *in vitro* and *in vivo* HCV infection systems and increased understanding of HCV biology have led to the development of many HCV-specific small molecules with antiviral activity. There is new optimism in HCV treatment programs with the recent completion of Phase III studies of several protease inhibitors [3]. However, the potential for HCV mutants that escape from these direct-acting antivirals is a source of concern. Additional approaches are clearly needed for treatment and prevention of infection. An effective HCV vaccine has yet to be achieved, despite considerable effort. A required step in the design of a preventive vaccine for HCV is to identify relevant mechanisms of immune protection. For HCV, emerging evidence indicates a protective role for virus-neutralizing antibodies. Animal studies showed that protection from an infectious HCV inoculum with HCV-specific IgG is correlated with antibody titers blocking infection of target cells with pseudotyped retroviral particles expressing HCV E1E2 glycoproteins (HCVpp) [4]. Other studies with HCVpp observed a relationship between the control of virus infection and the neutralizing antibody response in single source outbreaks of acute HCV infections [5], [6]. In addition, antibodies to HCV E2 prevent infection in a murine model with a chimeric human liver [7], [8]. Finally, a recently developed immunocompetent humanized mouse model for HCV exhibited a robust antibody response to a recombinant vaccinia virus expressing HCV C-E1-E2-p7-NS2 proteins that protected from a heterologous infectious HCV challenge in some of the animals, and correlated with the serum level of antibodies to E2 [9]. Consequently, understanding the antibody-epitope interaction provides a basis for the formulation of a B cell-based vaccine to prevent HCV infection.

The HCV envelope glycoprotein E2 is a major natural target for a protective antibody response, although there are a number of limitations. First, a significant challenge is defining conserved epitopes in this highly diverse virus that are capable of eliciting protective antibodies. HCV is classified into seven major genotypes with more than 30% divergence between genotypes, and each genotype containing a large number of related subtypes that differ between 20–25% at the nucleotide and amino acid (aa) level [10], [11]. The virus replicates at a high rate and exists in an infected individual as a swarm of quasispecies [12]–[14]. A rapid rate of quasispecies formation contributes to the emergence of viral variants escaping immune containment. A major region of variability is the first hypervariable region (HVR1) located at the N-terminus of E2. While HVR1 contains highly immunogenic epitopes that induce neutralizing antibodies, they tend to be isolate-specific, leading to viral escape [15]–[17]. Second, not all antibodies to E2 mediate virus neutralization. We previously described a large panel of human monoclonal antibodies (HMAbs) to HCV E2 [18], [19]. Cross-competition analysis segregated these antibodies into three immunogenic clusters with all of the non-neutralizing antibodies falling into one cluster, designated as antigenic domain A [19], [20]. Isolation of these antigenic domain A antibodies indicated that they are similar to the non-neutralizing Fabs isolated by phage display [21], and to the non-neutralizing serum antibodies present in individuals with chronic HCV infections [22]. Third, it has been proposed that a segment of E2 encompassing aa434–446, “epitope-II," contains epitopes that are associated with non-neutralizing antibodies; more importantly, these antibodies interfere with the neutralizing activities of antibodies directed at an adjacent E2 segment encompassing aa410–425 [23], [24]. Fourth, specific N-glycans on E2 negatively modulate neutralizing antibodies to E2, perhaps by interfering with their binding to nearby contact residues that are part of their epitopes [25]–[28].

Nonetheless, substantial progress has been achieved in identifying broadly neutralizing monoclonal antibodies that are directed at the CD81-binding site. Some of these antibodies are against linear epitopes within aa412–423 [29]–[31], but the majority of these antibodies are against conformational epitopes on E2 [18], [27], [32]–[38]. Cross-competition analysis has revealed that many of these neutralizing HMAbs are directed at overlapping epitopes, which can be grouped into two distinct clusters, and both clusters mediate neutralization by inhibiting E2 binding to CD81 [19], [27]. One of these clusters, designated as antigenic domain B, contains antibodies displaying varying degrees of broad neutralizing activities against different genotype and subtype HCVpp [27], [39], [40]. Epitope mapping studies revealed two E2 residues at G530 and D535 that are required for binding by all antigenic domain B HMAbs [27], [39], [40]. Similar studies with other broadly neutralizing HMAbs also recognize epitopes containing G530 and D535 [36]–[38]. Importantly, the residues G530 and D535 are absolutely conserved and shown to participate in the interaction of E2 with CD81 [41], [42]. Thus, antigenic domain B antibodies broadly neutralize different HCV isolates by competing with CD81 for binding to conserved residues on E2 that are important for viral entry. A key question for vaccine development is whether immune selection by some antigenic domain B antibodies can lead to virus escape. This possibility is suggested by a study of virus neutralization by antigenic domain B antibodies against a sequential panel of HCV variants of a single patient with chronic HCV infection [17]. Some of these antibodies showed varying degrees of neutralization against the variants from different time points. Others showed sustained neutralization against the majority of these variants. Furthermore, we recently reported on three patterns of virus escape under immune pressure by propagating cell culture infectious virus, 2a HCVcc, under increasing concentrations of a neutralizing antibody [43]. Of the three tested antigenic domain B antibodies, one antibody led to escape mutant viruses without affecting viral fitness. A second led to escape but with compromised viral fitness and a third led to complete virus elimination without escape mutants. These findings collectively highlight the rarity of viral epitopes that are both conserved and not associated with virus escape.

To identify highly conserved epitopes that will be relevant for vaccine design, we used the information gained from epitope mapping of antigenic domain A and B HMAbs to construct soluble E2 mutants that do not bind to their respective antibodies. By employing yeast surface display of antibodies, HCV HMAbs were isolated that initially bound to a 1a HCV E2 antigenic domain A mutant to minimize the selection of non-neutralizing HMAbs and then bound to a second 1a HCV E2 antigenic domain B mutant to minimize the selection of antigenic domain B HMAbs. We describe in this report a panel of nine HMAbs to overlapping epitopes on HCV E2, designated as HC-84-related antibodies. Each HMAb neutralized HCVcc of different genotypes with varying profiles and potency, and mediated neutralization by inhibiting E2 binding to CD81. Epitope mapping revealed distinct contact residue patterns that differ from antigenic domain B. More importantly, when infectious HCVcc was co-cultured with each of the tested HC-84 antibodies, virus escape was not observed.

## Results

The majority of neutralizing antibodies to conserved conformational epitopes inhibits E2 binding to CD81. Epitope mapping of these antibodies, which we designated as antigenic domain B, revealed two contact residues located at aa530 and 535. We suspected that among the antigenic domain B antibodies, only a few epitopes are invariant due to structural or functional constraints [43]. To isolate novel antibodies, we developed an algorithm employing modified E2 antigens that led to the identification of a novel cluster of neutralizing antibodies to E2.

### Isolation of non-antigenic domain A and B human monoclonal antibodies

A yeast display scFv antibody library was constructed from peripheral blood B cells obtained from an individual with asymptomatic chronic HCV genotype 2b infection. The donor was identified after testing nearly 90 different sera from HCV seropositive blood donors for binding and neutralizing antibody titers. The donor's serum contained a high antibody binding titer (>1∶10,000) to E2 and similarly high neutralizing titers against genotype 2a JFH1 HCVcc and 1a H77C HCVpp. Immunoglobulin heavy chain variable (VH) and light chain variable (VL) gene regions from total RNA were amplified and cloned into the yeast vector pYD2 to generate a scFv-expressing yeast display library. The final library size was 2×10^7^ individual clones. Inserts of the correct size were found in 100% of 20 tested clones by PCR and showed 90% diversity by DNA sequence analysis.

**Figure 1:**
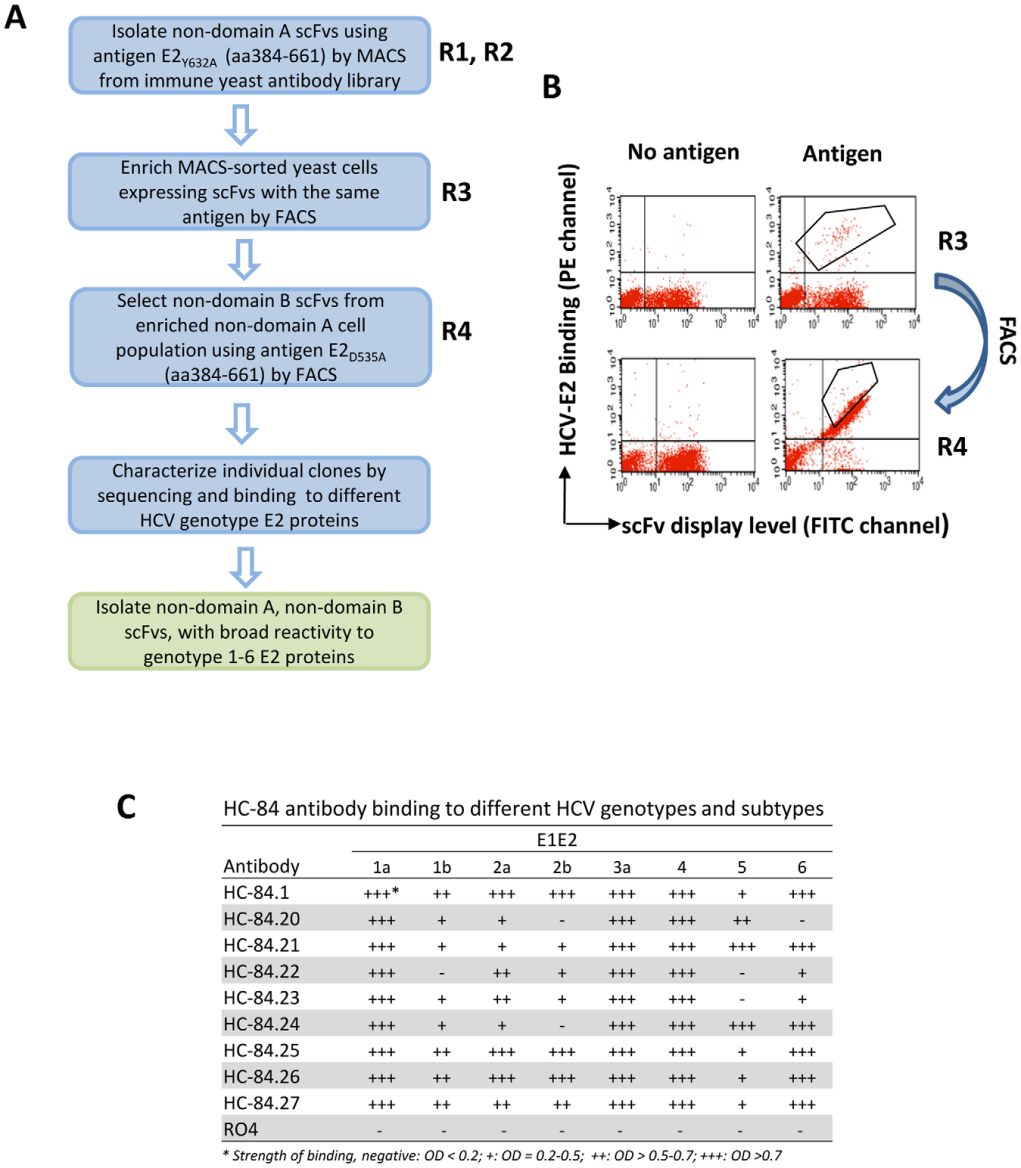
Isolation of novel human monoclonal antibodies to HCV E2. (**A**) Schematic diagram of the algorithm used to identify novel scFvs to E2 glycoprotein. (**B**) Cell sorting of magnetic bead-isolated scFvs (after R2) to select R3 and then to select R4. To select R3: 10^7^ MACS isolated cells (from R2 and designated as non-antigenic domain A cell population) were incubated with the same E2 proteins (E2_Y632A_) as used in R1 and R2, and then incubated with anti-V5 and HC-33.1 (an anti-E2 HMAb to a defined epitope [45]), at 10 µg/ml. The cells were then labeled with FITC-anti-mouse and PE or APC-anti-human IgG (Fcγ-specific) as described in Materials and Methods. The labeled cells, 10^7^ cells/ml, were used for sorting by flow cytometry. The sorting gates were set to collect the desired double positive cells. The “no antigen" control provided guidance on setting the sorting gates. To select R4: 5×10^6^ R3-collected yeast cells were incubated with E2_D535A_ after induction. Cell sorting was performed as described for R3 by FACS. (**C**) Binding to HCV genotypes and subtypes 1–6 recombinant E1E2 lysates by ELISA. The data represent the mean binding (optical density (OD) value) from three experiments. Each antibody was tested at 20 µg/ml. R04 is a negative control isotype-matched HMAb to HCMV.

HCV E2 mutant glycoproteins were designed for the selection of novel HCV HMAbs from the immune library. We have shown that HCV E2 contains at least three antigenic domains with neutralizing antibodies to overlapping conformational epitopes segregating into two antigenic domains (designated as antigenic domain B and antigenic domain C) and non-neutralizing antibodies to overlapping epitopes in one domain (antigenic domain A) [19], [20], [44]. Epitope mapping revealed that distinct but partially overlapping sets of amino acids are critical to the binding of antibodies within antigenic domain B [43]. Similar information was obtained for antigenic domain A antibodies (not published) [19], [20], [44]. This information led us to engineer E2 mutants to avoid selecting HMAbs to antigenic domain A and B epitopes by substituting with alanine a shared residue in each antigenic domain. Two E2 antigens for antigenic domain A and B knockouts were constructed by introducing mutations at respective positions Y632A and D535A (designated as E2_Y632A_ and E2_D535A_). The algorithm for isolating novel HMAbs is summarized in Figure 1A. Two rounds of magnetic immunobead, MACS, selection, R1 and R2, were performed to avoid selection of non-neutralizing anti-HCV HMAbs (antigenic domain A) and antibodies not specific to HCV E2. The yeast display scFv library was incubated with soluble E2_Y632A_ protein immobilized on the immunobeads and then separated. A third round of antigenic domain A depletion was again carried out with soluble E2_Y632A_ protein and the bound yeast cells were separated by fluorescence-activated cell sorting (FACS) (collected cells are as indicated in R3, Figure 1B). E2 bound by scFvs was detected by HC-33.1, an HMAb to an epitope located on E2 in a region encompassing amino acids 410–425 [45]. Correctly displayed scFvs on yeast surface were detected by anti-V5 against the SV5 tag. The next round of FACS selection was performed to deplete antigenic domain B scFv expressing yeast cells by incubating the collected non-antigenic domain A fraction with soluble E2_D535A_ protein and selecting bound scFvs by FACS (collected cells are as indicated in R4, Figure 1B). A total of 300 monoclonal scFv yeast cells were screened for binding activity to HCV E2. Fingerprint and DNA sequence analyses from the isolated scFv yeast cells identified 75 unique scFv (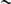
25%). Each monoclonal yeast cell bound to HCV E2 but not to a no-antigen control. We next tested the ability of these scFvs to bind to soluble E2 derived from six different HCV genotypes and subtypes, 1a (H77C), 1b (UKN1B5.23), 2a (UKN2A1.2), 2b (UKN2B2.8), 3a (UKN3A1.9), 4 (UKN4.11.1), 5 (UKN5.15.7) and 6 (UKN6.5.8). A final nine scFvs that showed a broad breadth of reactivity and had unique sequence combinations of heavy and light chain CDR1, 2 and 3 regions (data not shown) were converted to full IgG_1_ molecules and transiently expressed (Figure 1C). These HCV HMAbs are designated as HC-84.1, HC-84.20, HC-84.21, HC-84.22, HC-23, HC-84.24, HC-84.25, HC-84.26 and HC-84.27. Each antibody was tested against a panel of recombinant cell-associated E1E2 proteins (the same panel used to derive soluble E2 from six different HCV genotypes and subtypes) by ELISA (Figure 1C). Each HC-84 HMAb bound broadly, with HC-84.1, -.21, -25, -.26 and -27 binding to all isolates.

### Breadth of neutralization and inhibition of E2 binding to CD81

**Figure 2:**
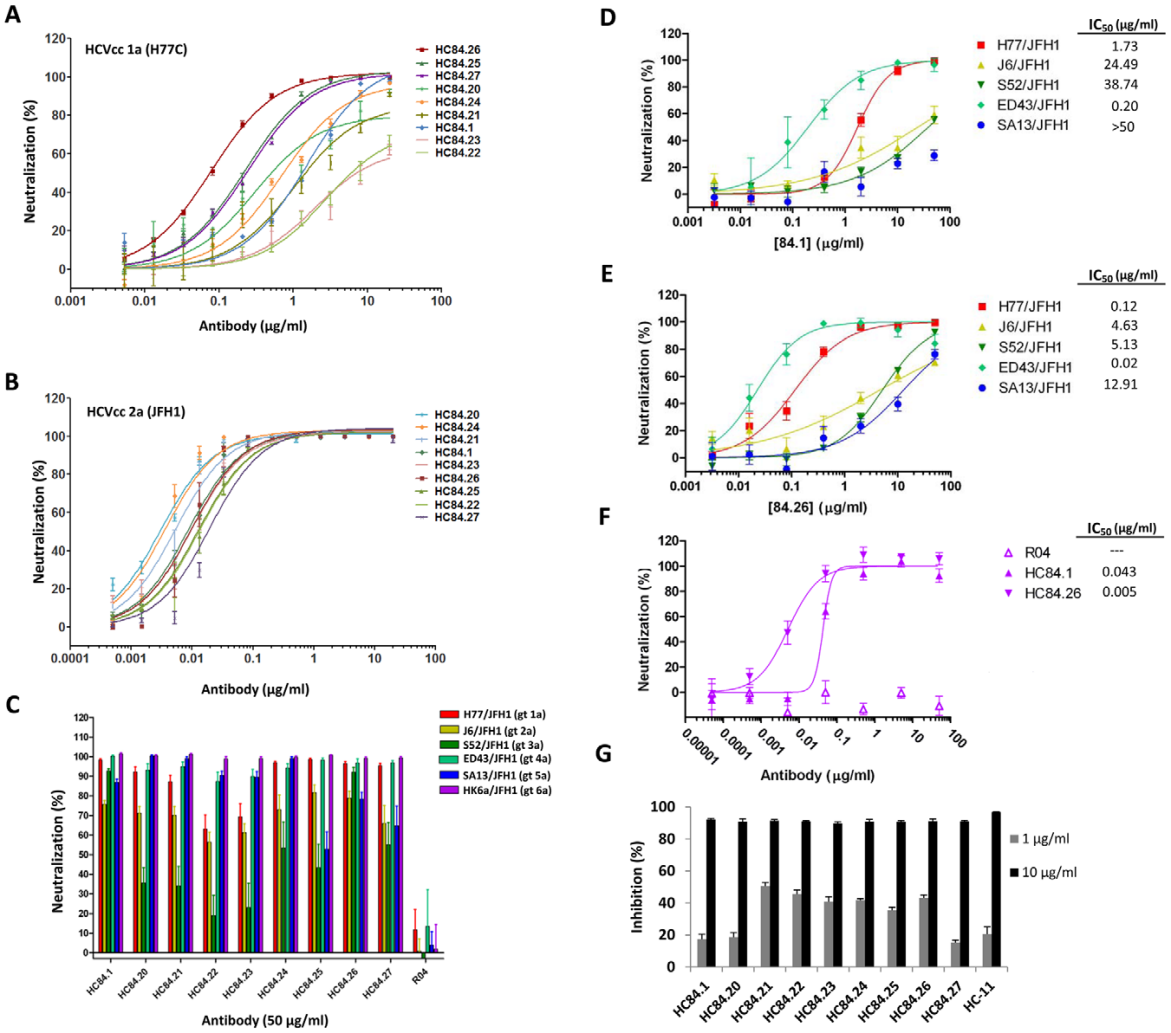
Breadth of neutralization and inhibition of E2 binding to CD81 by HC-84 HMAbs. (**A**) Dose-dependent neutralization of 1a H77 HCVcc and (**B**) 2a JFH1 HCVcc, as determined by FFU reduction assay [27], [43]. The antibodies are ordered from the lowest to the highest concentration required to reach 50% maximal neutralization concentration (IC_50_), as summarized in Table 1. Infectious 1a H77C (HJ3–5) chimeric virus or 2a JFH1 inoculum was incubated with each HMAb, at concentrations ranging from 0.005–20 µg/ml against 1a H77C and from 0.0005–20 µg/ml against 2a JFH1, prior to inoculation onto Huh7.5 cells that were pre-seeded in eight-well tissue culture chamber slides. Cells were fixed and immunostained with a MAb to NS3 antigen at day 4 p.i., and enumerated by FFU-reduction assay. Each assay was performed in triplicates and data are shown as percent neutralization, the mean of three experiments ±SD. (**C**) Neutralization of JFH1-based genotypes 1–6 C-NS2 recombinant viruses as determined by FFU reduction [60]–[62]. The designation of the viruses are: genotype 1a (H77C/JFH1), 2a (J6/JFH1), 3a (S52/JFH1), 4a (ED43/JFH-1), 5a (SA13/JFH1) and 6a (HK6a/JFH1) [11], [60], [63], [64]; all except the 2a virus contained adaptive mutations. R04 is an isotype-matched HMAb negative control. Infectious virus inoculum was incubated with each HMAb at 50 µg/ml followed by inoculation onto Huh7.5 cells. Cells were immunostained with a MAb to NS5A antigen at 45 hrs p.i., and enumerated by FFU. The error bars are SEMs of 8 replicates (from 4 replicates each in 2 independent assays) compared with 12 replicates of virus only (from 6 replicates each in 2 independent assays). (**D–E**) Dose-dependent neutralization of JFH1-based genotypes 1–5 C-NS2 recombinant viruses by (**D**) HMAb HC-84.1 and (**E**) HC-84.26 were determined by FFU reduction assay. IC_50_ values for each respective antibody against different genotype HCVcc are as indicated. (**F**) Dose-dependent neutralization of HC-84.1 and HC-84.26 against JFH1-based genotype 6a recombinant virus by FFU reduction assay. R04 is an isotype-matched HMAb negative control. IC_50_ values for each antibody are as indicated. Infectious virus inoculum was incubated with each HMAb at 0.005–50 µg/ml (in **D** and **E**) or 0.00005–50 µg/ml (in **F**) followed by inoculation onto Huh7.5 cells. Cells were immunostained with a MAb to NS5A antigen at day 2 p.i., and enumerated by FFU. The error bars are SEMs of 4 replicates compared with 6 replicates of virus only. (**G**) Inhibition of E2 binding to CD81-LEL by HC-84 HMAbs. Genotype 1a H77C recombinant E1E2 lysate containing 1 µg/ml E2 was incubated with each test HMAb at 1 and 10 µg/ml, and the antibody-antigen complex was then added onto CD81-LEL-precoated wells. Detection of E2 bound to CD81-LEL was measured with biotinylated CBH-4D [18]–[20]. HC-11 was used as a positive control. The experiments were performed twice in triplicate. Error bars indicate one standard deviation from the mean.

Having implemented a bias-screening approach to select for novel neutralizing antibodies, we next investigated the neutralizing activities of these HMAbs (Figure 2). The purified IgG_1_ HMAbs at 20 µg/ml were first tested for neutralization by FFU reduction assay against 1a H77C and 2a JFH1 HCVcc to assess neutralization activity and whether they were directed to conserved epitopes. Each HMAb neutralized both HCVcc isolates (data not shown). Dose-dependent neutralization from 0.005–20 µg/ml was performed against 1a H77C HCVcc and from 0.0005–20 µg/ml against 2a JFH1 HCVcc, and ranked (in the figures) based on the concentration required to reach 50% neutralization, IC_50_, as calculated by nonlinear regression (Figures 2A and 2B). As summarized in Table 1, HC-84 HMAbs neutralized 1a HCVcc with IC_50_ ranging from 0.08–272 µg/ml and neutralized 2a HCVcc with IC_50_ ranging from 0.003–0.020 µg/ml. The panel of antibodies was further tested against a panel of 2a JFH-1 chimeric HCVcc bearing C, E1, E2, p7 and NS2 from genotypes: 1a (strain H77C), 2a (J6), 3a (S52), 4a (ED43), 5a (SA13), and 6a (HK6a) at 50 µg/ml (Figure 2C); all except the 2a virus contained adaptive mutations. All nine HMAbs neutralized 1a, 2a, 4a, 5a and 6a HCVcc by >40%. The only isolate in which no neutralization was observed with some of the antibodies (as defined by <40% neutralization) was against the genotype 3a HCVcc. HC-84.1, -.24, -.25, -.26 and -.27 showed >40% neutralization. The remaining antibodies, HC-84.20, -.21, -.22 and -.23 showed <40% neutralization. R04, an isotype-matched HMAb to HCMV, exhibited no neutralization. IC_50_ values against all genotypes were determined with HC-84.1 and -.26 since these two antibodies showed more uniform neutralization (Figures 2D–2F). For HC-84.1, the IC_50_ ranged from 0.043 µg/ml (against HK6a/JFH1) to >50 µg/ml (against SA13/JFH1) (Figures 2D and 2F). HC-84.26 IC_50_ values ranged from 0.005–12.91 µg/ml (Figures 2E and 2F). R04, as expected, showed no neutralization (not shown, except in Figure 2F). Of note is the different sensitivity to neutralization between the two genotype 2a isolates, JFH1 and J6 (Figures 2B and 2E). For both HC-84.1 and HC-84.26, J6/JFH1 HCVcc required higher levels of antibody concentrations than against JFH1 to achieve similar degrees of neutralization (Figures 2B and 2E, Table 1). Since the titration studies against different HCVcc genotypes were performed with a second batch of HC-84.1, both batches were compared against the genotype 3a isolate (Figure S1). The IC_50_ for the first and second batches were respectively 4.3 and 15 µg/ml. A possible explanation is the extent of IgG aggregation between the two batches, which could affect antibody function and stability. Overall, broad patterns of neutralization were observed with the entire panel of HC-84 HMAbs. Differences between breadth of neutralization and breadth of binding to E2 genotypes and subtypes (Figure 1C) could be due to different binding affinities or isolates employed for the respective studies. We next examined whether HC-84 HMAbs mediated neutralization by inhibiting the binding of E2 to CD81. As shown in Figure 2G, pre-incubation of 1a H77C E2 glycoproteins with 1 and 10 µg/ml of each HC-84 HMAb reduced E2 binding to CD81 in a dose-dependent manner, as observed with a control antigenic domain B antibody, HC-11 [27]. Over 90% inhibition of E2 binding to CD81 was achieved with each antibody at 10 µg/ml. The neutralization activities observed with each of the HC-84 HMAbs demonstrated that they are not antigenic domain A antibodies. The employment of E2_Y632A_ efficiently avoided the selection of non-neutralizing antibodies.

**Table 1:** Neutralization potency of HC-84 antibodies.

Antibody IC50 (µg/ml)									
HCVcc	HC-84.1	HC-84.20	HC-84.21	HC-84.22	HC-84.23	HC-84.24	HC-84.25	HC-84.26	HC-84.27
**1a**	1.52±0.12	0.30±0.05	0.88±0.29	2.72±1.09	1.79±0.84	0.68±0.11	0.23±0.046	0.08±0.00	0.24±0.02
**2a**	0.009±0.001	0.003±0.000	0.005±0.001	0.014±0.005	0.010±0.001	0.004±0.000	0.013±0.004	0.010±0.004	0.020±0.001

### HC-84 HMAbs bind to conformational epitopes that are not within antigenic domains A and B

**Figure 3:**
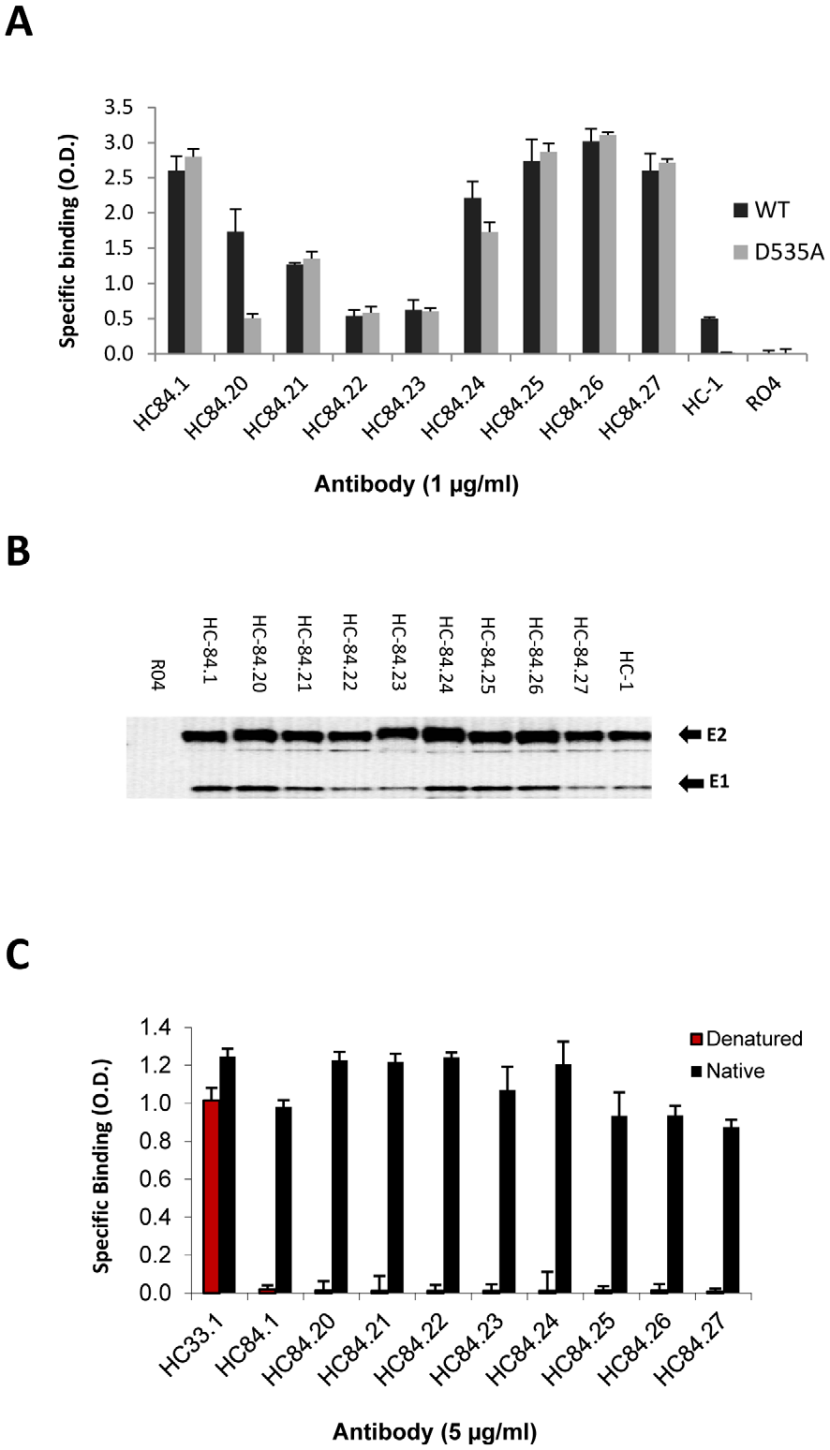
HC-84 HMAbs bind to conformational epitopes that are not within antigenic domains A and B. (**A**) Antibody binding to H77C (wt) and D535A recombinant E2 lysates by ELISA. The assays were performed with 1 µg of E2/ml that was captured by GNA pre-coated wells, and followed by incubation with each HMAb at 1 µg/ml (*x*-axis). Positive control is HC-1, an antigenic domain B HMAb [27] and negative control is R04. The *y*-axis shows the mean optical density (O.D.) values. Data are derived from triplicate wells, the mean of two experiments ±SD. (**B**) Immunoprecipitation of 1a H77C recombinant E1E2 lysate by each HCV-84 HMAb (as indicated at the top of the panel). HC-1, an antigenic domain B HMAb, was used as a positive control and R04 was used as a negative control. The immunoprecipitated pellet was separated by sodium dodecyl sulfate-10% polyacrylamide gel electrophoresis under reducing conditions, and immunoblots were analyzed with HMAbs recognizing linear epitopes: anti-E2, HC-33.1 [45] and anti-E1, H-111 [49]. (**C**) HC-84 HMAbs do not bind to denatured 1a HCV E2. Recombinant E1E2 lysate was either left untreated (black bars) or denatured by incubation with 0.5% sodium dodecyl sulfate and 5 mM dithiothreitol for 15 min at 56°C (red bars). After treatment, the proteins were diluted 1∶5 in BLOTTO and captured by pre-coated GNA wells. After washing and blocking, bound proteins were incubated with each HC-84 HMAb at 5 µg/ml (*x*-axis) and a control HMAb, HC-33.1 [45]. Bound antibody was detected as described in Materials and Methods. The *y*-axis shows the mean optical density values for triplicate wells, the mean of two experiments ±SD.

To evaluate the efficiency of the bias-screening algorithm to avoid selecting antigenic domain B antibodies, we tested the binding of each HMAb to recombinant cell-associated wt H77C E2 and E2_D535A_ mutant by ELISA (Figure 3A). As expected, the nine HC-84 HMAbs bound to both E2 proteins nearly equally except for HC-84.20, which showed significantly greater binding to E2_D535A_ (0.51 OD) compared with HC-1(0.02 OD) or R04 (0.01 OD) binding to E2_D535A_ (*p*<0.0001), but with 70% reduction compared to binding against wt E2 (1.74 OD, Figure 3A), suggesting that HC-84.20 may partially overlap with antigenic domain B antibodies. As is typical of antigenic domain B antibodies, HC-1 [27] bound to wt E2 and not to E2_D535A_ since D535 is a contact residue. R04 showed no binding to both E2 proteins. The employment of E2_D535A_ in the screening algorithm led to the successful isolation of novel broadly neutralizing HCV HMAbs. All nine antibodies were able to immunoprecipitate recombinant 1a H77C E1E2 from cell lysate (Figure 3B). A slower E2 migration pattern was observed for HC-84.23 and HC-84.24, which is likely due to gel distortion by the heavy chains of immunoglobulin. Treatment of the E1E2 by heating to 56°C in the presence of 0.5% SDS and 5 mM DTT resulted in the complete abrogation of reactivity for all nine HC-84 HMAbs by ELISA, indicating that these antibodies target conformational epitopes on HCV E2 (Figure 3C). HC-33.1, an antibody directed to a mostly linear epitope on the E2 glycoprotein, was used as a positive control, and as expected retained 80% binding to E1E2 after denaturation (Figure 3C) [45]. We next measured the binding affinity of the HC-84 HMAbs by employing purified scFv of each antibody as determined by surface plasmon resonance in a BIAcore 3000 (Table 2, Figure S2). Purified 1a H77C secreted E2 was first captured onto a pre-coated sensor chip with CBH-4D, an antigenic domain A HMAb to a conformational epitope [18]–[20]. This step was taken to ensure that native E2 was employed in these measurements since only a fraction of the total purified E2 is functionally active because of the intrinsic deficiency of secreted E2 produced by overexpression in mammalian cells, which includes misfolding, aggregation, and different degrees of glycosylation [46]. Eight of nine HC-84 HMAbs were successfully expressed as scFvs. Figures S2A1–S2A8 show the overlay plots of association and dissociation curves for each of the scFvs to obtain *K*_on_, *K*_off_, and *K*_D_ values (Table 2). The ranked order, from the highest-affinity antibody, HC-84.23, to the lowest-affinity antibody, HC84.1, is within a narrow range of no greater than 6-fold difference. This is in contrast to a wider range in IC_50_ values against 1a HCVcc for these antibodies (Table 1). In addition, there was no correlation between affinity and neutralization potency suggesting that both affinity and specificity influence the neutralization activities of anti-HCV HMAbs.

**Table 2:** *K*_D_ measurements for E2 binding by HC-84 antibodies.

	*K*_on_ (M^−1^ s^−1^)	*K*_off_(s^−1^)	*K*_D_(M)
**HC-84.23**	2.95e04	1.00e-05	3.39e-10
**HC-84.20**	1.36e05	5.42e-05	3.99e-10
**HC-84.21**	3.47e04	2.74e-05	7.90e-10
**HC-84.22**	2.98e04	1.20e-04	4.03e-09
**HC-84.27**	8.26e04	3.50e-04	4.24e-09
**HC-84.25**	9.76e04	4.31e-04	4.42e-09
**HC-84.26**	9.03e04	4.33e-04	4.80e-09
**HC-84.1**	5.91e04	3.80e-04	6.43e-09

### Epitope mapping of HC-84 HMAbs

**Figure 4:**
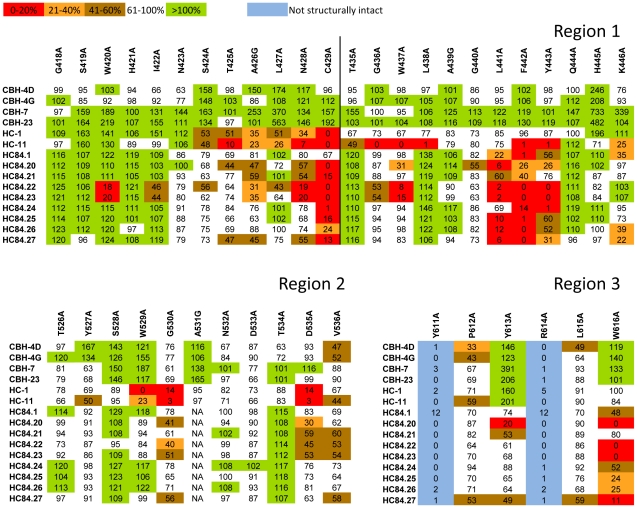
Epitope mapping. E2 mutant proteins were expressed in 293 T cells and cell lysates were analyzed by ELISA. Each HC-84 HMAb was tested at 2 µg/ml. Individual protein expression was normalized by binding of CBH-17, an HCV E2 HMAb to a linear epitope [18]. Three regions of E2 protein were analyzed: Region 1 encompassing aa418–429 and aa435–446, Region 2, aa526–536, and Region 3, aa611–616. Red indicates 0–20%, orange 21–40%, brown 41–60%, white 61–100% and green >100% binding when the residue was replaced by alanine (or glycine at aa426, 439, 531), relative to binding to wt. Blue indicates not structurally intact E2 conformation, as defined by control antibodies, CBH-4D, -4G, -7, -23, HC-1 and -11. Retention of binding by these antibodies is necessary to ensure native E2 structure. Data are shown as mean values of two experiments performed in triplicate.

To assess which of the contact residues bound by the HC-84 HMAbs participate in binding to CD81, epitope mapping was performed by site-directed alanine substitution studies in defined E2 regions (Figure 4). Three separate segments of E2 encompassing aa418–446, aa526–536 and aa611–616 (respectively designated as regions 1, 2 and 3 in Figure 4) were selected based on previous observations that residues within these regions are essential for E2 binding to CD81 [41], [42], [47], [48]. A series of alanine substitution H77C E1E2 mutants covering the three regions were constructed by site-directed mutagenesis. Binding by each of the HC-84 HMAbs to these mutants was measured by ELISA using lysates of transiently transfected HEK293 cells. The results were normalized according to the E2 abundance in each lysate, as determined by the binding of a non-neutralizing HMAb, CBH-17, directed at a linear E2 epitope [18]. To confirm that the E2 conformational structure was not altered with each alanine substitution, binding by antibodies representing antigenic domain A (CBH-4D and -4G) and antigenic domain C (CBH-7 and -23) was also measured, since they have minimal to no cross-reactivity to antigenic domain B antibodies [44]. Thus, a substitution resulting in reduced binding to the test antibody and to either or both antigenic domain A and C antibodies was interpreted as having a global effect on E2 structure instead of being specific for the test antibody. Two antigenic domain B antibodies, HC-1 and HC-11, were included in this analysis to determine the extent of overlap between antigenic domain B and HC-84 HMAbs [27].

**Figure 5:**
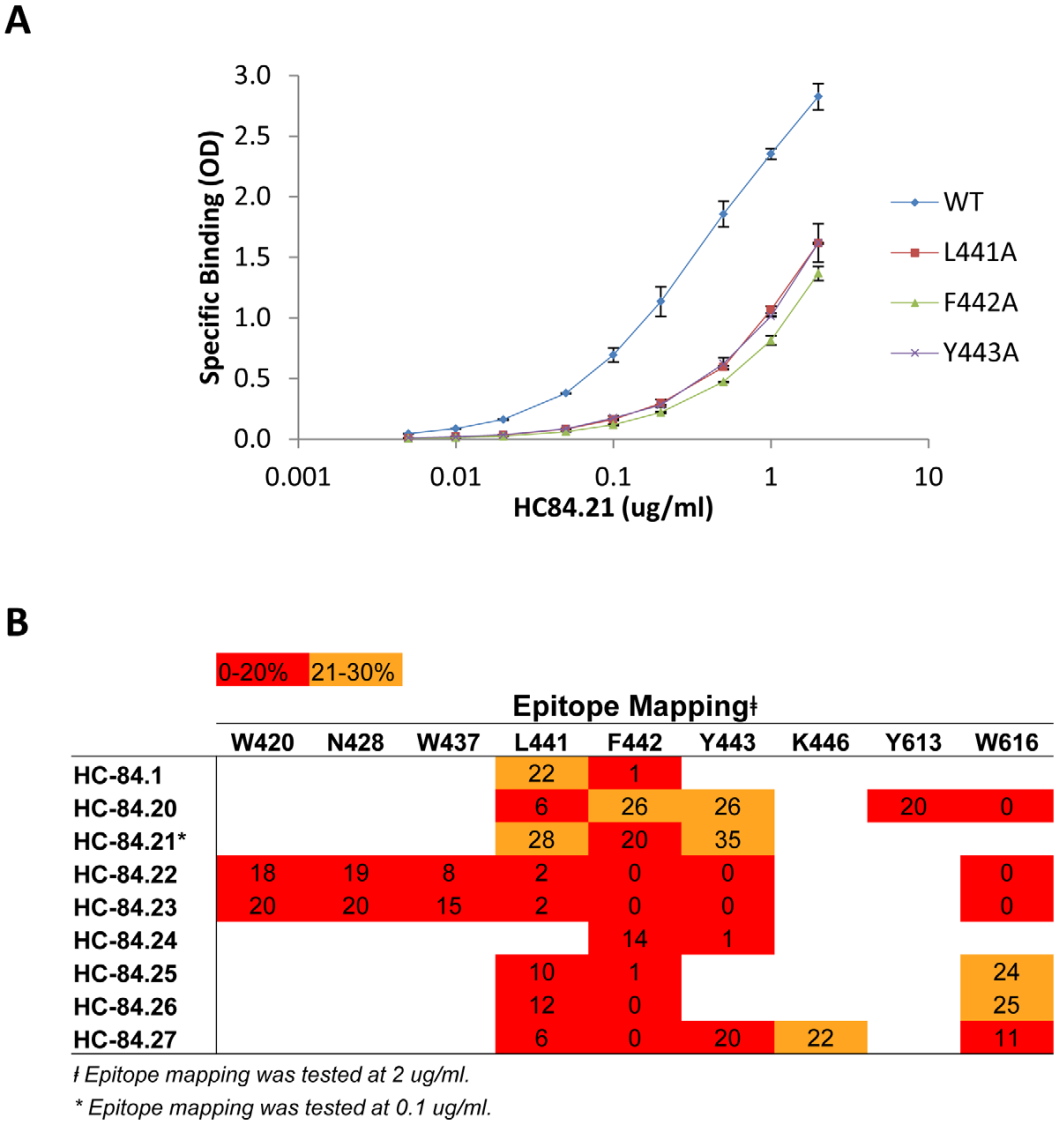
Epitope location for each HC-84 antibody. (**A**) HC-84.21 dose-dependent binding, 0.005–2 µg/ml, was measured by ELISA against E2 mutants bearing alanine substitutions at L441, F442 or Y443, and wt H77C. Data are shown as mean optical density (O.D.) values ±SD of two experiments performed in triplicate. (**B**) Summary of epitope location for each HC-84 antibody, as shown in Figure 4. Contact residues for HC-84.21 are based on antibody binding at 0.1 µg/ml in Figure 5A. Red indicates 0–20%, orange 21–40%.

As shown in Figure 4, alanine scanning of the three regions of E2 revealed that eight residues located at aa420, 428, 437, 441, 442, 443, 613 and 616, within regions 1 and 3, bound ≤20% relative to wt by at least one of the HC-84 HMAbs (when tested at 2 µg/ml), which indicates that these residues are involved in their respective epitopes. Note that binding to the cysteine at aa429 is discounted as a contact residue since a substitution at this site would be expected to have significant structural effects. The exception was HC-84.21, which showed relative to wt >20% binding at all of these sites. Since HC-84.21 has similar binding and neutralization profiles, and substantial cross-competition with other HC-84 antibodies (data not shown), a dose-dependent study employing 0.005–2 µg/ml was performed against three E2 mutants containing alanine substitutions at aa441, 442 or 443 (Figure 5A). Substantial difference in binding compared to wt was observed at each concentration. At 0.1 µg/ml, binding to aa441, 442 and 443 mutants was respectively 23%, 17% and 25% of wt, suggesting that these residues participate in the HC-84.21 epitope. As a cluster, the HC-84 epitopes are centered at aa441, 442 and 443 with the majority including a contact residue at aa616 (Figure 5B). The >70–80% reduction in binding observed with each HC-84 HMAb to the identified residues in Figure 5B (compared to binding to wt) was confirmed with testing at 0.1 µg/ml (data not shown). While <20% binding retention indicate involvement as a contact residue, binding retention between 21–30% suggests probable involvement, as shown in Figure 5B. Two of the antibodies, HC-84.22 and -.23 also contain contact residues at aa420, 428 and 437. Epitope mapping of HC-11 revealed contact residues (<20% binding) located at aa425, 428, 436, 437, 438, 442, 443, 530 and 535 (Figure 4). The shared contact residues between HC-84 antibodies and HC-11, an antigenic domain B antibody, are aa428, 437, 442 and 443, which indicate a high degree of overlap between antigenic domain B and the HC-84 antibodies. It remains possible that other HC-84-related contact residues could be identified by evaluating the E2 alanine substitution library (as shown in Figure 4) at a lower antibody concentration (0.1 µg/ml) since testing at 2 µg/ml is higher than the *K*_d_ values for most of these antibodies. Nonetheless, HC-84 epitopes are distinctly separate from antigenic domain B epitopes. Moreover, our findings confirmed the validity of the screening algorithm employing designed E2 mutants to identify novel antibodies.

Sequence alignment of the nine amino acids encompassed by the HC-84 epitopes (as identified in Figure 5B) with other HCV sequences in an HCV database (http://hcv.lanl.gov) revealed that six residues located at aa420, 428, 441, 443, 613 and 616 are 100% conserved in all genotypes and subtypes. For the remaining three residues, sequence analysis showed that residue W437, specific to the epitopes of HC-84.22 and -.23, was 100% conserved in genotype 1 and over 99% conserved in genotype 4. A different side chain change, F437, is observed in <1% of genotype 4. However, F437 is dominant in genotypes 2, 3, 5 and 6, which suggests that a mutation at residue 437 could lead to escape from neutralization by HC-84-related antibodies. Residue F442, shared in all of the HC-84 epitopes, is 100% conserved in genotypes 1, 2, 3 and 4 but M442 or L442 are found at a low frequency in genotypes 5 and 6. Residue K446, unique to the epitope of HC-84.27, was 100% conserved in genotypes 1, 3 and 4, but not in genotypes 2, 5 and 6. Overall, the sequence of each residue within the HC-84 epitopes has remained highly conserved among HCV genotypes and subtypes.

### Effects of each HC-84 contact residue on virus entry and binding to CD81

**Figure 6:**
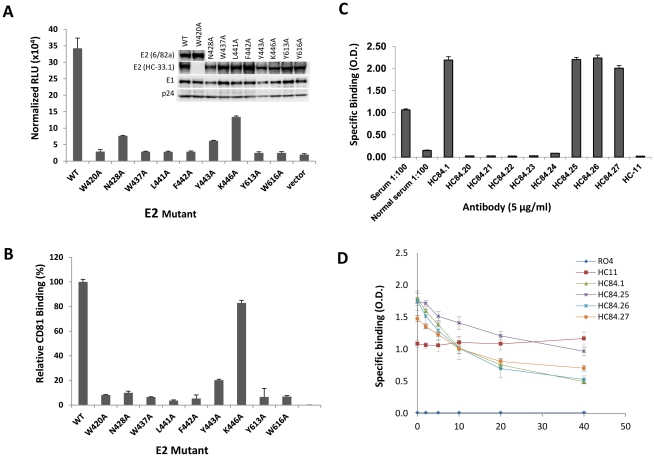
Effect of alanine substitution with each HC-84 contact residue on HCVpp entry, interaction with CD81, and HC-84 HMAb binding to “epitope-II". (**A**) Wt and H77c HCVpp mutants, each bearing a single alanine substitution at a contact residue of HC-84 HMAbs, were produced, normalized by p24 as described in Materials and Methods, and employed to infect Huh7.5 cells. Virus entry was assessed by measuring luciferase activity on day 3 p.i. Results are shown as luciferase activity signals in infected cells, relative to signals from cells infected with vectors alone. Data are shown as the mean of three experiments ±SD, with each performed in triplicates. The inset shows incorporation of E1E2 glycoproteins into wt and mutant HCVpp that were partially purified by pelleting virus through a 20% sucrose cushion and followed by Western blot analysis. E2 in the majority of HCVpp mutants and wt were probed with HMAb HC-33.1, and in W420A HCVpp and wt, were re-probed with MAb 6/82a [29]. E1 was probed with HMAb H-111, an antibody to a linear E1 epitope [49]. HIV p24 was probed with an anti-HIV p24 antibody as a loading control. The images are composites. (**B**) Effect of each HC-84 contact residue on E2 binding to CD81. HC-84 epitope- related E2 mutant proteins were expressed in 293 T cells and captured in microtiter plates by GNA. Individual protein expression was normalized by binding of CBH-17, an HCV E2 HMAb to a linear epitope [18]. The wells were then incubated with CD81-LEL at 20 µg/ml. Detection of CD81-LEL captured by wt or mutated E2 was measured with anti-CD81 and shown as percent CD81 binding relative to wt. Data are derived from triplicate wells and shown as the mean of two experiments ± SD. (**C**) HC-84 HMAb binding to epitope-II by ELISA. Biotinylated-epitope II, aa434–446, was captured by streptavidin in microtiter wells. The wells were then incubated with each HC-84 HMAb at 10 µg/ml, serum (1∶100 dilution) from the individual whose B cells were employed to isolate the HC-84 antibodies, and a normal human serum (1∶100 dilution), as a negative control. Specific binding was detected by anti-human IgG-labeled horseradish peroxidase. (**D**) Peptide (epitope-II) inhibition of HC-84 antibody binding to E2. Recombinant H77C E1E2 lysate was captured by GNA in microtiter wells. The wells were then incubated with selected HC-84 HMAbs and two controls, HC-11 and R04 that were pre-incubated with epitope-II (labeled as peptide) at indicated concentrations. Binding was detected after anti-human IgG-labeled horseradish peroxidase. For C and D, the *y*-axis shows the mean optical density values for triplicate wells, the mean of two experiments ±SD.

To begin to assess the structural and functional constraints for entry of each contact residue within the HC-84 epitopes, virus infectivity was measured using mutant 1a H77C HCVpp bearing an alanine substitution at each of the contact residues of HC-84 HMAbs (Figure 6A). Over 90% reduction in viral entry compared to wt was observed with substitution at aa420, 437, 441, 442, 613 or 616, as measured by luciferase readout. This could imply a high degree of structural or functional constraint and was consistent with the observation that these residues were 100% conserved in all genotypes and subtypes, except for F442. However, in genotype 1a, F442 was 100% conserved. Substitution at aa428 and 443 led respectively to 78% and 82% reduction in viral entry. The alanine side chain replacement at these two sites also has significant structural or functional impact, although less than that of the absolutely conserved residues. The only E2 mutant that maintained moderate entry capacity, with 61% reduction relative to wt, had a substitution at aa446. This contact residue is restricted to HC-84.27. To rule out the possibility that the lost infectivity was caused by impaired E1E2 assembly affected by introduced mutations in the E2 protein, each mutant HCVpp was partially purified through a 20% sucrose cushion followed by Western blot analysis and probed for E1 and E2, and p24 to control loading levels for HCVpp (inset in Figure 6A). For the majority of HCVpp mutants and wt, the E2 proteins were probed by HC-33.1. Since the HC-33.1 epitope was known to contain a contact residue at W420 [45], the W420A HCVpp mutant, along with wt, were probed with MAb 6/82a that is directed at an H77C HVR1 epitope [29]. E1 was identified by H-111, an HMAb to an E1 linear epitope [49]. HIV p24 was identified by an anti-HIV p24 antibody as a loading control. The levels of E1 and E2 incorporated in the HCVpp mutants were similar to the levels observed in wild-type HCVpp. Since each of the amino acids within the HC-84 epitopes led to reduced HCVpp infectivity, their role in E2 binding to CD81 was assessed by a CD81-capture ELISA. Cell lysate of mutant 1a H77 HCVpp bearing an alanine substitution at each of the residues was captured onto GNA-coated microtiter plates. The amount of E2 captured by GNA was normalized by binding to CBH-17. CD81-LEL at 20 µg/ml was then added and binding to CD81 was detected by an anti-CD81 monoclonal antibody (Figure 6B). Relative CD81 binding to each E2 mutant compared to wt was <10% for each HC-84 contact residue except for the substitution mutants at aa428 (10%), 443 (20%) and 446 (83%). The three E2 mutants with substitutions at aa428, 443 or 446 retained >10% entry capacity compared to wt, and showed ≥10% retention in binding to CD81. There was a general correlation between reduction in viral entry, as measured by relative HCVpp infectivity, and reduction in binding to CD81.

### HC-84 HMAb binding to an E2 peptide encompassing aa434–446

A segment of E2 encompassing aa434–446 (“epitope II") has been proposed to include residues involved in the epitopes of non-neutralizing antibodies that interfere with the neutralizing activities of antibodies directed at an adjacent E2 segment encompassing aa412–426 (“epitope I") [23], [24]. The presence of interfering antibodies could be a significant negative modulator of neutralizing antibodies and is highlighted by the detection of antibodies to epitope II in four of nine serum samples from patients with chronic HCV infection [23], [24]. Since all of the HC-84 epitopes contain contact residues at L441, F442 or Y443, and two of the epitopes also contain contact residues at W437, direct binding assays of each HC-84 HMAb to a biotin-linked synthetic peptide encompassing aa434–446 of H77C E2 was performed (Figure 6C). Four of the nine antibodies, HC-84.1, -.25, -.26 and -.27, at 5 µg/ml bound strongly to aa434–446 (O.D.>1.0). The presence of serum antibody to aa434–446 of the donor whose B cells were employed to isolate the HC-84 HMAbs was also tested and found to have significant binding at a 1∶100 dilution. A serum from a normal blood donor (seronegative to HCV) and HC-11, an antigenic domain B HMAb, showed no binding to the synthetic peptide. To confirm the direct binding of HC-84.1, -.25, -.26 and -.27 to aa434–446, non-biotinylated aa434–446 was used to inhibit the binding of HC-84 antibodies to recombinant 1a E1E2 lysate (Figure 6D). The synthetic peptide from 2 µg/ml to 40 µg/ml progressively diminished the binding of each of the tested HC-84 antibodies, and not of HC-11. However, maximum inhibition of binding was at 50%. Since these antibodies are to conformational epitopes, it would be expected that a synthetic peptide would not be able to completely inhibit the binding of these antibodies to native E2. Collectively, our findings showed that this region is the target of broadly neutralizing antibodies to HCV, although it remains possible that aa434–446 also could be recognized by interfering non-neutralizing antibodies to predominantly linear epitopes.

### The HC-84 epitopes resist escape mutations in a 2a HCVcc isolate

The *in vitro* selection of monoclonal neutralizing antibody escape mutants, by repetitive neutralization and passage of cell culture infectious HCV in the presence of the antibody, represents a powerful approach to mapping amino acid residues within the viral envelope that contribute to antibody binding [43], [50]. Our recent study on neutralization escape from three antigenic domain B antibodies revealed that these antibodies bind to at least two discontinuous regions of E2 encompassing aa425–443 and aa529–535 [43]. Furthermore, aa425–443 is a region of variability that is responsible primarily for viral escape from neutralization, with or without compromising viral fitness. The region aa529–535 is a core CD81-binding region that does not tolerate neutralization escape mutations. Identification of contact residues responsible for escape from HC-84 antibodies will clarify which residues within the E2 region encompassing aa425–443 are more invariant, and which residues when mutated lead to escape with or without a cost in viral fitness. We previously designed a viral escape selection protocol to maximize the likelihood of escape variants by subjecting wt HCVcc to increasing concentrations of the selection antibody starting at IC_50_ (µg/ml) value (Figure S3) [43]. At each antibody concentration, the extracellular virus was passaged repeatedly to reach a titer of 1×10^4^ FFU/ml before the virus was subjected to the next higher antibody concentration. This step allows minor variants to be amplified prior to the next round of selective pressure at a higher antibody concentration. As a control virus population, wt HCVcc was subjected to serial passages in increasing concentrations of R04 to provide reference viral variants. This permits specific discrimination between mutations introduced during long-term *in vitro* propagation of wt HCVcc and those mutations induced under the selective pressure of HC-84 antibodies. A second control was CBH-2, an antigenic domain B-neutralizing HMAb, which leads to viral escape without a cost in viral fitness [39], [43]. At each passage of extracellular virus, infected cells were monitored for virus escape by screening with a two-color indirect immunofluorescence assay (IFA) that uses both the test antibody, and a second antibody that recognizes virus replication regardless of a change in envelope antigenicity [43], [50]. In this case, we detected cells infected with an escape mutant by the loss of specific binding by the test antibody, but with retained binding by an anti-NS3 antibody. When escape was detected, RNA from escape mutants was extracted from either cells or culture supernatants, reverse-transcribed, PCR amplified, and subcloned. Genomic residues 1491–2579 spanning the entire E2 coding region were sequenced from selected individual clones. The number of clones that were sequenced and analyzed ranged from 20 to 60 per sample.

**Figure 7:**
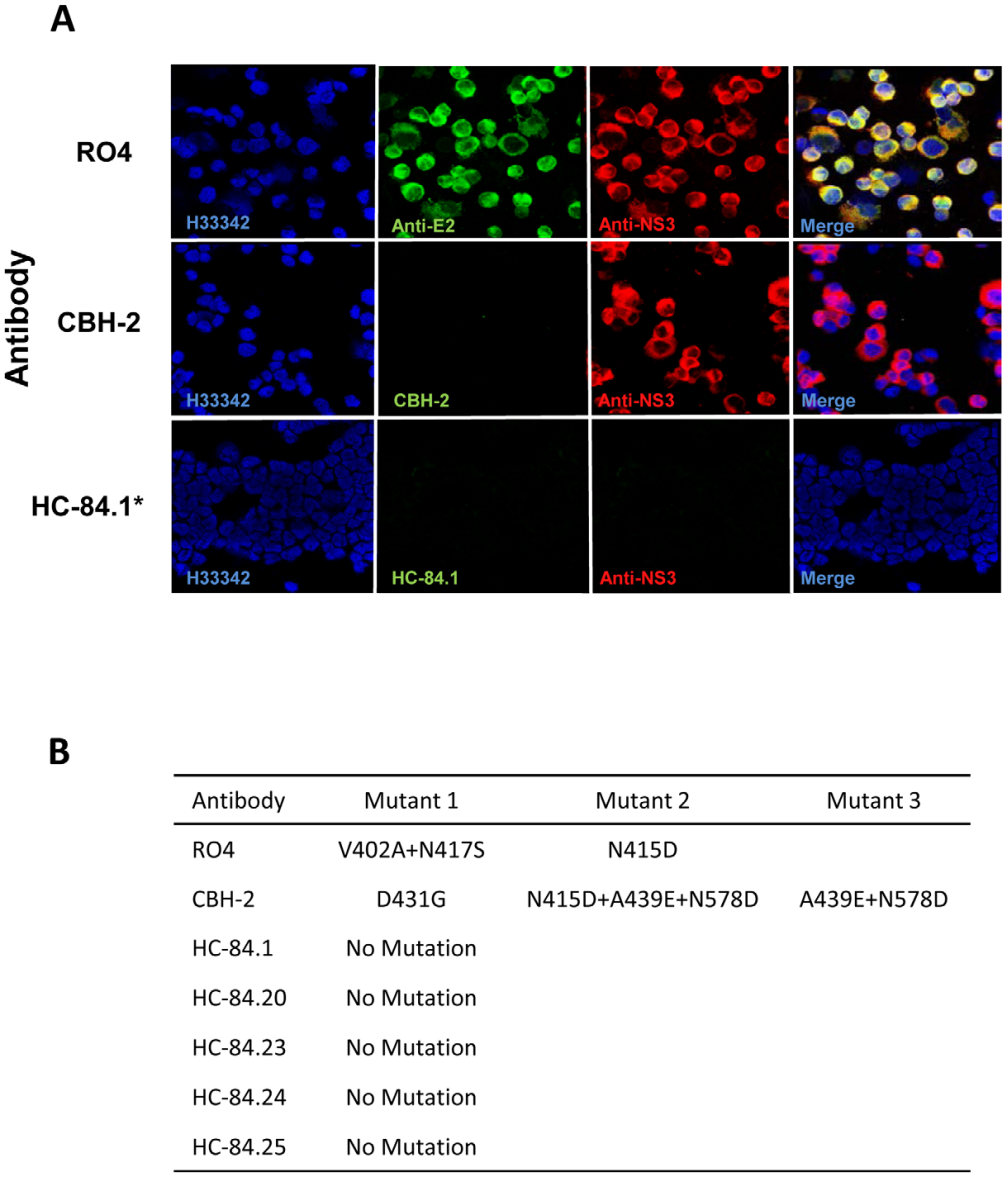
Identification of neutralization escape mutants and associated amino acid changes in the HCV E2 glycoprotein. (**A**) Dual antibody immunofluorescence staining of Huh7.5 cells infected with JFH1 2a virus after multiple rounds of neutralization by the respective antibody. R04, a human monoclonal antibody against CMV was used as mock selection. HCV E2 glycoprotein was stained with the respective antibody under which viral escape mutants were selected (green). Total virus infected cells were stained with anti-NS3 antibody labeled with Alexa-594 (red). The cells were counterstained with Hoechst nuclear stain H33342 (blue). Escape mutants were assessed for CBH-2 (a neutralizing domain B HMAb [43]) at passage 3 in 10 µg/ml, HC-84.1 at passage 4 in 0.5 µg/ml, HC-84.20 at passage 4 in 0.1 µg/ml, HC-84.23 at passage 1 in 10 µg/ml, HC-84.24 at passage 2 in 0.05 µg/ml and HC-84.25 at passage 5 in 0.5 µg/ml. *Only HC-84.1 is shown to represent the tested HC-84 antibodies. (**B**) Observed amino acid substitutions in neutralization escape mutants.

Five antibodies were selected for escape studies, HC-84.1, -.20, -.23, -.24 and -.25, three with (HC-84.20, -.23 and -.25) and two without (HC-84.1 and -.24) contact residues at aa616. In addition, the HC-84.23 epitope also includes W420 and N428. W616 is thought to be a contact residue involved in E2 binding to CD81 [47], [51]. The modest degree of viral entry of 16% and 21% observed respectively with 1a H77C HCVpp mutants with substitution at N428A or Y443A suggested that viral escape could be observed at these residues (Figure 5B). Figures 7A and 7B show neutralization escape profiles for the five tested antibodies, and the control antibodies, R04 and CBH-2. The concentration of R04 was raised rapidly since this antibody had no effect on HCV and only 1–2 passages at each antibody concentration were needed to reach 10^4^ FFU/ml HCVcc. Spontaneously formed variants containing V402A and N417S, or containing a single mutation at N415D, were identified (Figure 7B), as previously observed [43]. CBH-2 when increased progressively from 0.1 to 1 µg/ml required 3–4 passages before the virus reached a titer of 10^4^ FFU/ml. This was consistent with the antibody neutralizing a substantial portion of wt HCVcc. When the concentration increased from 1 to 10 µg/ml and after three passages at 10 µg/ml, 100% of the infected cells were not stained by CBH-2 but were stained by anti-NS3 (Figure 7A). Three sets of escape mutants were identified: a mutant with a single mutation at D431G, a mutant with triple mutations at N415D, A439E and N578D, and a mutant with double mutations at A439E and N578D (Figure 7B). These CBH-2 escape variants are identical to the ones previously observed and escape has been linked to mutations at aa431 and aa439 [43].

Under the conditions tested, selective pressure of each tested antibody, HC-84.1, -.20, -.23, -.24 and -.25, led to no escape in two independent experiments with 2a HCVcc. The lack of escape is shown for HC-84.1 (Figure 7A) to represent each of the tested HC-84 antibodies. At low antibody concentrations of <0.05 µg/ml, up to four passages at each concentration were required for the virus titer to reach 10^4^ FFU/ml. This indicated some degree of virus neutralization. When HC-84.1 reached 0.5 µg/ml, after four passages in HC-84.1, the virus was completely eliminated. A similar pattern of virus elimination was observed with HC-84.25, after five passages at 0.5 µg/ml of HC-84.25. HC-84.20 eliminated the virus after four passages at 0.1 µg/ml. HC-84.24 was the most efficient; virus elimination occurred at 0.05 µg/ml after two passages. HC-84.23 was the least efficient. After eight passages in 0.5 µg/ml, rare infected cells (<1%), detected by both HC-84.23 and anti-NS3, could still be observed but the virus titer could not be increased. The rare infected cells persisted as the concentration was increased from 1 to 5 µg/ml, even though the virus titer was <10^4^ FFU/ml. After two passages at this antibody concentration, some of the rare infected cells were not stained by HC-84.23 but were stained by anti-NS3, which indicates the possibility of escape. When the concentration was increased to 10 µg/ml, no infected cells were observed corresponding to virus elimination. The concentration associated with virus elimination indicated the following order of potency: HC-84.24>HC-84.20>HC-84.1=HC-84.25>HC-84.23. This order of potency is in rough agreement with the narrow range of IC_50_ for these antibodies against 2a HCVcc (Table 1), in which the potency ranking is HC-84.20>HC-84.24>HC-84.1>HC-84.23>HC-84.25. We attempted to rescue the virus by passaging cultured supernatant when infected cells were not observed with each of the HC-84 HMAbs onto naïve Huh7.5 cells in the absence of respective antibody for two additional passages, and no detectable virus emerged from the passaged supernatants. The failure to generate antibody-induced HCV escape mutants could be due to the viral strain employed in these studies. However, it is also possible that the selected antibodies are to highly conserved epitopes such that each contact residue for them is essential and the induction of escape within this epitope leads to a lethal change in virus function or structure.

### HCV E2 structure analysis

**Figure 8:**
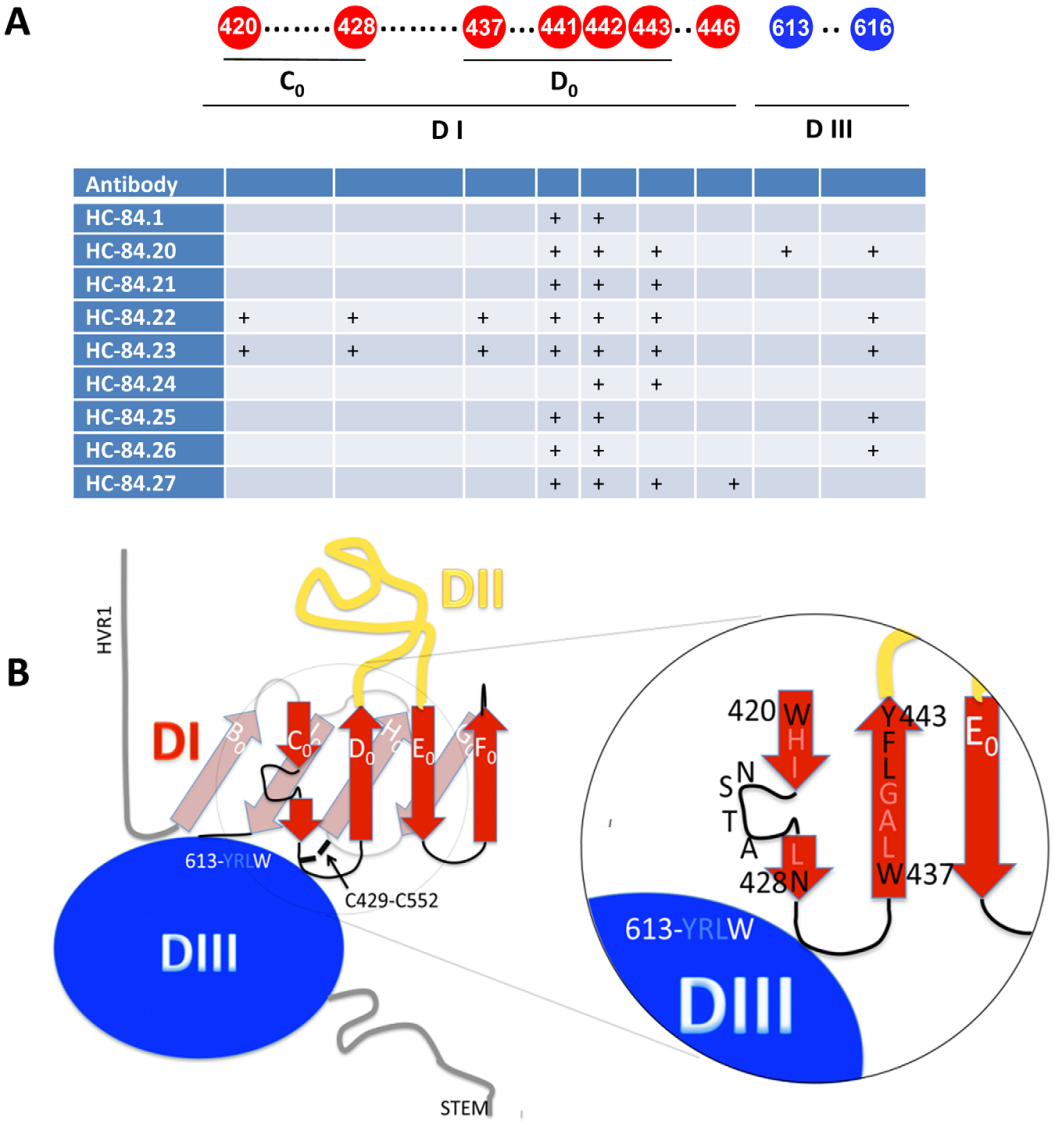
HCV E2 structural analysis. (**A and B**) Comparative analysis of the nine HC-84 antibody epitopes and their location on a structure model of the E2 glycoprotein. Contact residues (shown in circles) are located in two discontinuous regions on E2, aa420–446 and aa613–616. Red indicates residues in domain I; black is a cysteine; and blue is residues in domain III. The specific contact residues for the HC-84 antibodies (+) span two domains (I and III) of a structural model of E2, within the central domain I the residues are located on two β-strands, C_0_, and D_0_. (**B**) Mapping of the HC-84 contact residues on the N-terminal side of the 4-stranded C_0_D_0_E_0_F_0_ β-sheet and more specifically, the C_0_D_0_ β-hairpin. To account for the distance between residues 420 and 428, the glycosylation site at 423–425 is shown to be bulging out and with the loop connecting the C_0_ and D_0_ strands having a somewhat convoluted 3-dimensional conformation. Residues 428 and 437 are brought close in space in the proposed model.

A tertiary model for HCV E2 was recently proposed, based on the 3-domain, β-sheet-rich “class II" fold of the flavivirus fusion proteins [52]. This model took into account the HCV E2 disulfide bonds, the residues that are part of the CD81 binding site and the data on deletion mutants of E2 that still bind CD81 and conformational antibodies [53]. Mapping the alanine scanning results of the HC-84 HMAbs on this model indicates that their corresponding epitopes are on the exposed surface of domain I (DI) and also cover a small region of domain III (DIII) (Figure 8A). More precisely, in domain I the residues highlighted in this work map to the N-terminal side of the 4-stranded C_0_D_0_E_0_F_0_ β-sheet (i.e., the C_0_D_0_ β-hairpin, Figure 8B). Both of these regions (in domains I and III) are also part of the CD81 binding site, suggesting that both the receptor and the HC-84 antibodies bind at the interface between these two domains. In the viral class II fusion proteins for which structures are available, domains I and III are close in space in the pre-fusion form. Being along a β-strand, it is likely that W420 and I422 are buried (the sequence is 419-SWHIN-423, alternating buried hydrophobic and exposed hydrophilic residues, the hallmark of a β-strand), and that a mutation at W420A results in a local distortion of the conformation, such that the conformational antibodies binding to other epitopes are not affected. This is likely to also be the case for the 441-LFY-443 sequence, in which at least one residue (either L441 and Y443, or F442, depending on the register of the D_0_ β-strand) would be buried in the β-sheet and not exposed for direct contact with the antibody or with the CD81-LEL. Mutation of the concerned residue to alanine could induce only a local distortion at this epitope. Notably, replacement of F442 by tryptophan reduced HCVpp entry to 30% [41], [42], [47], [48], while an alanine at that position completely abrogated CD81 binding and HCVpp entry, further supporting the requirement for a large hydrophobic side chain at position 442. The HC-84 epitope mapping findings provide additional information to further refine the available model, since the distance between residues 420 and 428 would be about 24 Å, if they were along a single strand containing a glycosylation site (423-NST-425) in the middle. If residues 420 and 428 are part of the epitope, a long strand spanning the two would imply that mutating the residues in between would also affect binding, which is not observed. The new data would thus be more compatible with the glycosylation site bulging out, and with the loop connecting the C_0_ and D_0_ strands having a somewhat convoluted 3-dimensional conformation, than that suggested by the flat 2D diagram of the E2 model. The tertiary structure model brings residues 428 and 437 close in space, matching with the fact that C429 is disulfide-bonded with C552, which would be right underneath W428 in the opposite β-sheet (B_0_I_0_H_0_G_0_, facing the viral membrane, Figure 8B). This end of DI, opposite to DII, is likely to be in close contact with DIII, as illustrated in Figure 8, and the interaction with DIII may affect its conformation. This spatial arrangement in the DI-DIII interface may also be affected by the mutation of the DIII residues, which may not necessarily make physical contact with the antibody or with the CD81-LEL.

## Discussion

The HCV E2 glycoprotein encodes clusters of overlapping epitopes that are highly immunogenic with evidence that the more dominant epitopes do not elicit the most broadly protective antibodies. For example, the HVR1 region displays immunodominant epitopes that are mainly targeted by isolate-specific antibodies from which the virus is able to rapidly escape [15]–[17]. A second cluster of highly immunogenic epitopes, designated as antigenic domain B, contains overlapping conformational epitopes that account for the majority of the identified broadly neutralizing antibodies [18], [32]–[38]. Although antigenic domain B antibodies exhibit broad neutralization against different HCV genotypes and subtypes, it is probable that HCV is able to escape from immune pressure by the majority of these antibodies [43]. A third cluster, designated as antigenic domain A, includes epitopes that induce non-neutralizing antibodies [19], [20]. It is also probable that antigenic domain A and other non-neutralizing determinants are highly immunogenic and account for a substantial portion of antibody response to E2 [21], [22]. Taken together, HCV is able to divert the immune response to these highly immunogenic determinants and thereby gains a selective advantage.

We implemented a screening approach for novel antibodies that avoided these determinants. Heterologous E2 employed in the screening eliminated antibodies to HVR1. Information gained from epitope mapping of previously isolated HCV HMAbs led to the development of mutant E2 antigens that minimized the selection of non-neutralizing antigenic domain A antibodies and neutralizing antibodies to antigenic domain B. Based on broad binding patterns to different genotype and subtype E2 proteins, nine scFvs were selected for conversion to IgG_1_ and were further studied. Surprisingly, all nine antibodies mediated virus neutralization and neutralized both 1a and 2a HCVcc. Our earlier experiences in isolating HMAbs to HCV, using an initial screen by IFA binding to recombinant E2, yielded nearly 50% of the isolated antibodies that are non-neutralizing [18], [20]. A possible implication of this finding is that the majority of the non-neutralizing antibody response to HCV E2 is to antigenic domain A epitopes. If this proves to be the case, a vaccine candidate that avoids an antibody response to antigenic domain A could be an approach to focus the immune response to a repertoire of antibodies that eliminates at least a significant portion of non-neutralizing antibodies. As expected, all of these antibodies bound to a 1a D535A E2 mutant, which is not bound by broadly neutralizing antigenic domain B antibodies [27], [39], [40]. The nine HC-84-related antibodies, designated as antigenic domain D, broadly neutralized different HCVcc genotypes and subtypes, and many of these antibodies have greater neutralization potency against 1a and 2a HCVcc than two of the more potent antigenic domain B antibodies, HC-1 and HC-11 [27]. The IC_50_ values of antigenic domain D antibodies are substantially lower against 2a HCVcc (JFH1) than 1a HCVcc (H77) (Table 1). Subtle differences in the presentation of antigenic domain D epitopes between these two genotypes could account for the different IC_50_ values. Since these antibodies are derived from B cells of an individual infected with HCV genotype 2b, it is possible that the HC-84 HMAbs are more directed at their respective epitopes as presented in genotype 2. However, it is also possible that the JFH1 isolate is more sensitive to neutralizing antibodies than the H77 isolate. We previously reported on antigenic domain B antibodies isolated from B cells of an individual infected with genotype 1b having lower IC_50_ values against 2a HCVcc (JFH1) than 1a HCVcc (H77) [27]. Moreover, the IC_50_ values for HC-84.1 and -.26 against the 2a HCVcc (JFH1 and J6) isolates are significantly different. Even though their epitopes have complete sequence conservation between these two isolates, it is possible that global conformation with the variation (approximately 13%) in E2 glycoproteins of JFH1 and J6 could disturb antibody binding to their respective epitope. This, in turn, will be reflected in the observed differences in antibody-mediated neutralization. Additional studies will be required to link the amino acid(s) and precise location(s) that contribute to this possibility. Nonetheless, the patterns of neutralization against HCVcc of different genotypes (Figure 2C) suggest that these antibodies are directed at highly conserved epitopes. These antigenic domain D antibodies showed more uniform neutralization against different HCV genotypes and subtypes than antigenic domain B antibodies [40].

Based on the observation that each antigenic domain D antibody inhibits E2 binding to CD81, epitope mapping studies by alanine scanning focused on E2 segments encompassing aa410–446, aa526–540 and aa611–617. These regions have been reported to contain contact residues that form the E2 binding site to CD81 [41], [42], [47], [48]. As expected, contact residues were not located within aa526–540 but within aa410–446 and aa611–617. In the proposed model of the tertiary organization of HCV E2, domain I is organized such that β-strands C_0_D_0_, as well as E_0_ and F_0_, are consecutive in sequence, spanning aa418–444 and aa526–542 as two β-hairpins, respectively. While antigenic domain B antibodies are localized on the C_0_D_0_ and E_0_F_0_ β-hairpins, antigenic domain D antibodies are localized on C_0_D_0_ and on domain III. The location of antigenic domain D contact residues is in agreement with the model in which domains I and III are close in space. Although antigenic domain D is a distinct cluster of overlapping epitopes, there is some overlap between antigenic domain D and antigenic domain B. Some antigenic domain B antibodies, e.g., HC-11, share contact residues within C_0_D_0_, at residues 442 and 443. The HC-84 epitope mapping data makes possible several adjustments of the tertiary model to accommodate the distance between contact residues and the spatial orientation of a connecting loop between the C_0_ and D_0_ β-strands (Figure 8B). One adjustment involves the sequence aa441–443. This sequence was proposed to be located on β-strand D_0_, implying that at least one of the three residues would be buried in the β-sheet and not exposed to participate in binding to either CD81 or antibodies to this region, as discussed above. Of the three amino acids, L441 and Y443 are absolutely conserved but F442, although 100% conserved in genotypes 1, 2, 3 and 4, has low frequency changes to either M442 or L442 in genotypes 5 and 6. If F442 is buried and required for the structural integrity of the β-sheet, substitution with a large bulky residue like tryptophan is likely to distort the β-sheet less than substitution with a small side-chain like alanine. Interestingly, CD81 binding experiments showed that when phenylalanine was replaced by tryptophan, HCVpp entry was reduced by 70%, while for a F442A mutant no CD81 binding could be observed [48]. A second adjustment is that W420 and I422 may be buried in the β-sheet, such that a substitution at these residues provides only a local distortion. If that is the case, a question is the accessibility of these residues to participate in binding to CD81, as well as binding to antibodies directed at this region [41]. Moreover, these residues are absolutely conserved in the entire HCV database indicating functional or structural constraints preventing mutations at this site. Additional studies are required to confirm these modifications to the model. Overall, our findings provide support for this model, in which these antibodies bind to the same tertiary structure that interacts with CD81.

Since all of the antigenic domain D antibodies are affected by substitution to alanine at L441, F442 or Y443, we tested the binding of each antibody to a synthetic peptide encompassing aa434–446. This peptide has been proposed to encode non-neutralizing epitopes [23], [24]. However, several neutralizing monoclonal antibodies to linear epitopes in this region have been described [54], [55]. In our study, not only do the neutralizing antigenic domain D antibodies bind to contact residues within this sequence, but four of the nine antibodies, HC-84.1, -.25, -.26 and -.27, bound to this peptide indicating that the region aa434–446 forms an integral part of their epitopes. Although HC-84.25, -.26 and -.27 HMAbs are antibodies to conformational epitopes, their ability to bind to the aa434–446 synthetic peptide indicates that their epitopes contain a significant linear component. At the same time, binding by these antibodies to denatured E1E2 was significantly reduced (Figure 3C). One explanation could be that the synthetic peptide is sufficiently flexible to be shaped by the interaction with HC-84.1, -.25, -.26 and -.27 HMAbs leading to binding, but this cannot occur when the aa434–446 region is expressed in the context of denatured E1E2. Taken together, this region includes residues that are involved in conformational epitopes of potent and broadly neutralizing antibodies, although it remains possible that the E2 region aa434–446 encodes for non-neutralizing antibodies.

The initial expectation was for a minor subset of the antigenic domain D epitopes to be invariant because of functional or structural constraints. Surprisingly, when 2a HCVcc was grown in the presence of HC-84 HMAbs, under a viral escape selection protocol to maximize the likelihood of escape variants, five of five selected antibodies led to no escape variants, under the conditions tested. This can be explained in part by the findings in analysis of the effect of each contact residue within the HC-84 epitopes on H77C HCVpp entry (Figure 7A). Seven of ten contact residues, aa420, 429, 437, 441, 442, 613 and 616, when substituted with alanine led to >90% reduction in HCVpp entry compared to wt. Two other residues, aa428 and 443, when substituted reduced entry by approximately 80%. The only contact residue with moderate reduction was aa446, which is restricted to HC-84.27. This antibody was not selected for escape study. Reduction in entry correlated with reduction in binding to CD81 with each of these HC-84-related contact residues. The only residue when substituted without significant decrease in binding to CD81 was K446A. Previously studies have identified residues W420, W437, L441, F442, Y443, Y613 and W616 on E2 as participants in the interaction with CD81 [41], [42], [47], [48]. Substitution at these sites would be expected to negatively modulate entry. Substitution of the cysteine at aa429 also would be expected to alter the structure required for this interaction. However, it remains possible that escape from HC-84 HMAbs can occur. The leading candidate would be HC-84.27, which includes a contact residue at aa446. Mutations at this site would not significantly decrease virus entry. Although rare, mutations at W437 and F442 have been documented in the HCV database, but the mutations could be associated with a reduction in virus fitness. Moreover, since the JFH1 2a HCVcc is highly sensitive to antibody-mediated neutralization, escape studies with a less sensitive isolate will need to be performed to confirm our findings. Collectively, the HC-84 cluster of epitopes, designated as antigenic domain D, is highly conserved among HCV genotypes and subtypes, and mediates broad and potent virus neutralization that is not likely to lead to virus escape. Thus, these epitopes are relevant in vaccine design for this highly diverse virus.

## Materials and Methods

### Ethics statement

Ethical approval was obtained from Administrative Panel on Human Subjects in Medical Research (protocol number 13860), Stanford University, Stanford, California, USA. Written informed consent was obtained from the participant.

### Cell culture, antibodies, virus and reagents

HEK-293T cells were obtained from the ATCC. Huh7.5 cells (generously provided by Dr. C. Rice, Rockefeller University) were grown in Dulbecco's modified minimal essential medium (Invitrogen, Carlsbad, CA), supplemented with 10% fetal calf serum (Sigma-Aldrich Co., St. Louis, MO) and 2 mM glutamine. Yeast strain EBY-100 (GAL1-AGA1:URA3 ura3–52 trp1 leu2*Δ1 his3Δ200 pep4::HIS2 prb1Δ1.6R can1 GAL*) (Invitrogen, Carlsbad, CA) was maintained in YPD broth (Difco). HMAbs CBH-4D, CBH-4G, CBH-7, CBH-23, HC-1, HC-11, and H-111 against HCV E1E2 were produced as described [18], [19], [49]. HC-33.1 is an HMAb that binds to a mostly linear epitope between aa410–425 on the E2 glycoprotein [45]. MAb 6/82a against H77C HVR1 was generously provided by Dr. J. McKeating (University of Birmingham, UK). MAb against HCV NS3 protein was generously provided by Dr. G. Luo (University of Kentucky). MAb against human CD81 (clone JS-81) was purchased from BD Bioscience (San Jose, CA). MAb against V-5 tag was purchased from Invitrogen (Carlsbad, CA). The detection MAbs used in Fluorescence-activated cell sorting (FACS), namely Phycoerythrin (PE)-labeled donkey-anti-human IgG (Fcγ specific), FITC-labeled goat-anti-mouse IgG (Fcγ specific) and Allophycocyanin (APC)-conjugated donkey-anti-human IgG (Fcγ specific) were purchased from Jackson ImmunoResearch Laboratories (West Grove, PA). The cell culture infectious virus (HCVcc), 2a JFH1, was generously provided by Dr. T. Wakita (National Institute of Infectious Diseases, Japan) [56]. The 1a H77 HCVcc (HJ3–5) virus is an inter-genotypic chimeric virus produced by replacing the core-NS2 segment of the JFH-1 virus genome [56] with the comparable segment of the genotype 1a H77C recombinant [57]. A molecular clone encoding the CD81 large extracellular loop fused to glutathione *S*-transferase was generously provided by Dr. S. Levy (Stanford University) and affinity-purified over a GSTrap FF affinity column according to the manufacturer's instructions (GE Healthcare Bio-Sciences AB, Uppsala, Sweden). HCV E1E2 constructs, genotype (gt) 1b UKN1B5.23 (AY734976); gt 2a UKN2A1.2 (AY734977); gt 2b UKN2B2.8 (AY734983); gt 3a UKN3A1.9 (AY734985); gt 4 UKN4.11.1 (AY734986); gt 5 UKN5.15.7 (AY894682) and gt 6 UKN6.5.8 (EF427671) were generously provided by Dr. J. K. Ball (University of Nottingham). The yeast display vector pYD2 was kindly provided by Dr. J. D. Marks (UCSF) [58]. IgG_1_-Abvec for full-length IgG_1_ expression was kindly provided by Dr. P. Wilson (University of Chicago). Biotinylated peptides were synthesized using a C-terminal biotin residue with a gly-ser-gly linker (American Peptide, Sunnyvale, CA).

### Antigen preparation

Two 1a H77C E2 mutants were constructed containing either Y632A (E2_Y632A_) or D535A (E2_D535A_) substitution. H77C E2 (GenBank accession no. AF009606), aa384 to 661, was cloned into the expression vector pSec in-frame with the Igκ signal peptide sequence and fused with a myc and six-histidine tag at the carboxyl terminus. An alanine substitution was introduced at residue 632 or 535 using a QuikChange II site-directed mutagenesis kit (Agilent, La Jolla, CA) in accordance with the manufacturer's instructions. Mutations were confirmed by DNA sequence analysis (Sequetech, Mountain View, CA). The constructs were transfected into HEK293T cells via calcium-phosphate method and the supernatant was harvested after 5 days. E2 proteins were affinity purified over His-trap columns. The final products were more than 90% pure as judged by sodium dodecyl sulfate polyacrylamide gel electrophoresis (SDS-PAGE) analysis. The conservation of native E2 conformation of wild-type (wt) H77C E2 and the two mutants E2_Y632A_ and E2_D535A_ was confirmed using a panel of neutralizing and non-neutralizing HCV HMAbs to conformational epitopes on E2 by ELISA [27], [44].

### Generation of immune yeast antibody libraries

An immune library was constructed from peripheral blood B lymphocytes obtained from an asymptomatic individual infected with HCV genotype 2b infection. Total RNA, prepared from one million B cells, as previously described [18], was converted into cDNA using random hexamers. The cDNA products were used in primary PCR reactions to amplify the gamma heavy chain, kappa light chain and lambda light chain using the primers described elsewhere with the following modifications: for V_H_ primers, the sequences (5′-GT GGT GGTGGT TCT GCT AGC GGG GCC ATG GCC-3′ underlined is a *NcoI* site), (5′-ACC TCC GGA GCC ACC TCC GCC TGA ACC GCC TCC ACC TGT CGA CCC-3′ underlined is a *SalI* site) were added to the 5′ end of the forward and reverse primers respectively. For V_L_ primers, the sequences (5′-C GGT TCA GGC GGA GGT GGC TCC GGA GGT GGC GGA TCG -3′ underlined is a *BspE1* site), and (5′-GG GAT AGG CTT ACC TTC GAA GGG CCC GCC TGC GGC CGC-3′ underlined is a *NotI* site) were added respectively to all forward Vλ and Vκ primers, and all reverse Vλ and Vκ primers. In addition, a yeast display vector pYD2.A2 displaying A2-scFv was created by modifying the pYD2 vector, which comprises a [(Gly4-Ser)3] linker region carrying *SalI* and *BspEI* restriction sites and *NcoI* and *NotI* restriction sites flanking the inserted scFv. The pYD2 construct also contains an in-frame SV5 epitope. PCR-amplified heavy chain genes were pooled and cloned into vector pYD2.A2 using *NcoI* and *SalI*, yielding a heavy chain library of 5.0×10^6^clones, and the library was further digested with *BspE1* and *NotI* for gap repair with the light chain. In parallel, PCR-amplified light chain genes were pooled and ligated into vector pYD2.A2 using *BspE1* and *NotI*, yielding a light chain library of 5.0×10^6^clones. The V_L_ genes from the light chain library were re-amplified with primers HuJHF and Gap3 (HuJHF: 5′-ACC GTC TCC TCA GGG TCG ACA-3′, Gap3: 5′-GAG ACC GAG GAG AGG GTT AGG-3′). The resulting repertoire (10 µg) was then directly cloned into 50 µg *BspE*I- and *NotI*-pre-digested pYD2.A2.VH library through gap repair transformation into *Saccharomyces cerevisiae* strain EBY100. Library size was determined by plating serial dilutions of the transformation mixture on SD-CAA plates. This resulted in a yeast surface display of immune yeast antibody library of approximately 2×10^7^ clones. To validate the library, insert frequency and diversity were analyzed using colony PCR, DNA sequencing and SDS-PAGE/Western blot analyses.

### Selection of yeast display HCV E2-specific scFv

The yeast library was grown in SG-CAA for 48 hours at 18°C. Magnetic immunobead, MACS (Miltenyi, Auburn, CA), sorting was performed in accordance with the manufacturer's instructions. For the first two MACS selection rounds (R1 and R2), 2×10^9^ yeast cells were incubated with E2_Y632A_ (aa384–661) at 4°C for 20 min before being loaded onto a pre-treated column containing 25 µl of anti-myc microbeads. The column was then washed, followed by elution of bound yeast cells with 7 ml of SDCAA media using a plunger to push the cells out of the column, and then centrifuged at 2500× g for 5 minutes. The pellet was re-suspended and amplified in SD-CAA, followed by induction in SG-CAA. For the third round (R3) by FACS selection, 1×10^7^ MACS output cells (designated as non- antigenic domain A cell population) were incubated with the same E2 proteins (E2_Y632A_) at 4°C for 30 min in FACS wash buffer and then washed in cold wash buffer. The cells were then incubated with anti-V5 (1∶5000, Invitrogen) and HC-33.1 (an anti-E2 HMAb to a defined epitope [45]), at 10 µg/ml for 1 hr at 4°C. The anti-V5 against the SV5 tag was employed to verify correctly displayed scFv on yeast surface. HC-33.1 was employed to detect bound E2 on yeast surface. This was followed by another incubation with FITC-anti-mouse (1∶200) and PE- or APC-anti-human IgG (Fcγ specific) for 30 minutes at 4°C in the dark. The labeled cells were washed and re-suspended in FACS wash buffer at 1×10^7^ cells/ml for sorting by flow cytometry. Selection was performed using a BD Bioscience FACS Vantage Sorter and the sorting gates were set to collect the desired double positive cells. Collected cells were grown in SD-CAA medium and used for the next round of sorting after induction in SG-CAA, as described above. For the fourth round by FACS selection (R4), yeast cells (5×10^6^) were incubated with E2_D535A_ (aa384–661) (designated as non-antigenic domain B cell population). The cell sorting was performed as described above for the third round by FACS selection. After the final selection, collected cells were plated on SD-CAA plates and grown at 30°C for ∼2 days, after which individual clones were picked, induced, incubated with E2 proteins, and followed by detection with anti-E2 and anti-V5 antibodies. Double-positive clones were first analyzed by fingerprint. The scFv fragment amplified by PCR was digested with *BstNI* for 1 hour at 60°C. The reactions were separated on 2.5% agarose gel. The different banding patterns were analyzed and grouped. Selected clones representing each unique group were then sequenced to identify unique antibody sequences using primers PYFD and PYDR (PYDFor: 5′-AGT AAC GTT TGT CAG TAA TTG C-3′; PYDRev: 5′-GTC GAT TTT GTT ACA TCT ACA C-3′). The PCR product was then gel-purified and sequenced with primer GAP5 (Gap 5: 5′-TTA AGC TTC TGC AGG CTA GTG-3′).

### Production of scFvs and IgG_1_ HMAbs

To produce soluble scFvs, genes encoding scFvs were cloned from pYD2 into pSYN1 (a gift from Dr. J.D. Marks, UCSF), an expression vector imparting a c-myc and a hexahistidine tag at the COOH terminus. After isopropyl β-D-1-thiogalactopyranoside (IPTG) induction for overnight at 30°C, bacterial cells were harvested by centrifugation, resuspended in 200 mg/mL sucrose, 1 mM EDTA, 30 mM Tris-HCl (pH 8.0) on ice for 30 min, and centrifuged again to collect the supernatant. The pellet was resuspended in 5 mM MgSO_4_ on ice for 30 min and centrifuged to collect the supernatant. Both supernatants were pooled and loaded on a Ni^+^-NTA column preequilibrated with 30 mM imidazole/PBS and washed with 30 mM imidazole/PBS. Bound scFvs were eluted with 250 mM imidazole/PBS, dialyzed against PBS, and analyzed by SDS-PAGE, spectrophotometry and Bio-Rad RC DC protein assay (Bio-Rad, 500-0121). Conversion of scFv to full-length IgG_1_ was performed essentially as described [59]. In brief, V_H_ and V_L_ genes were PCR-amplified using primers to restore the human framework and append restriction sites. The resulting fragments were cloned into Igγ, Igκ or Igλ mammalian expression vectors containing the signal peptide and constant region genes. IgG_1_ was expressed by co-transfection of 293 T cells and cultured in serum-free medium. The expression levels were measured by ELISA and the resulting IgG_1_ were purified using proteinA affinity chromatography [18]. Purity and integrity of the HMAbs were analyzed by reducing and non-reducing SDS-PAGE.

### HCVcc neutralization assay by focus-forming unit reduction

Neutralization activities of HMAbs against different HCVcc genotypes were evaluated as previously described [43], [60]–[62]. Briefly, for neutralization experiments performed with H77C and JFH1 HCVcc, a virus inoculum (containing 50 FFU) was incubated with serial dilutions of antibodies for 1 hr at 37°C before inoculation onto Huh-7.5 cells (3.2×10^4^cells/well) that were seeded 24 hrs previously into 8-well chamber slides (Nalge Nunc, Rochester, NY). After 3 hrs of incubation at 37°C in the presence of 5% CO_2_, the inoculum was replaced with 400 µl of fresh complete medium followed by incubation for an additional 72 hrs. Infected cells were fixed and examined for NS3 protein expression by immunofluorescence detection of foci. The entire well was visualized in approximately 16 non-overlapping fields to obtain the number of foci. Each experiment was performed in triplicate. The antibody concentrations (µg/ml) causing 50% reduction in FFU (IC_50_) were determined by linear-regression analysis (GraphPad Software). The percent neutralization was calculated as the percent reduction in FFU compared with virus incubated with an irrelevant control antibody. All assays were performed in triplicate. For neutralization experiments performed with a JFH1-based genotype 1–6 HCVcc panel, a virus inoculum (∼100 FFU) were incubated for 1 h at 37°C with 50 µg/ml specific HMAbs prior to 3 h incubation with 6×10^3^ Huh7.5 cells/well in poly-D-lysine-coated 96-wells plates (Nunc). Cells were fixed and immunostained against NS5A 48 h post-infection [11], [60], [63], [64]. For each test, neutralization was done in eight replicates with 12 control wells containing the virus only, performed in two separate experiments by two investigators. Percentage neutralization was calculated in relation to the mean of virus only controls. Titration studies to calculate IC_50_ against genotype 1–6 HCVcc were similarly performed with selected antibody but with four replicates and 6 control wells containing only the virus (GraphPad Software).

### HCV-pseudotype retroviral particle production, infection and neutralization assay

HCVpp were produced as described [20], [65] by co-transfection of 293 T cells with pNL4-3.Luc.R^−^E^−^ plasmid containing the *env*-defective HIV proviral genome and an expression plasmid encoding the HCV glycoproteins or mutant E1E2 proteins. For the neutralization assay, the virus-containing medium was incubated with each HMAb at various concentrations, or phosphate-buffered saline instead of the antibodies as an infectivity control, plus 4 µg/ml polybrene at 37°C for 60 minutes [66], [67]. The HCVpp-antibody mixture was transferred to Huh7.5 cells (8×10^3^ cells/well) pre-seeded in 96-well plates, and infections were centrifuged at 730×*g* for 2 hrs at room temperature. After incubation at 37°C in the presence of 5% CO_2_ for 15 hrs, the unbound virus was replaced with fresh complete medium, followed by additional incubation for a total of 72 hrs. The neutralizing activity of an antibody was calculated as the percent reduction of luciferase activity compared with an inoculum containing phosphate-buffered saline (PBS). For HCVpp infectivity studies, the virus-containing extracellular medium was normalized for HIV p24 expression using a QuickTiter lentivirus titer kit (Cell Biolabs, San Diego, CA). All assays were performed in triplicate.

### Measurement of scFv affinity

ScFv binding kinetics were measured using surface plasmon resonance in a BIAcore 3000 (Pharmacia Biosensor) and used to calculate the *K_D_*. Approximately 135,000 response units (RU) of CBH-4D, an anti-E2 HMAb to a conformational epitope [18]–[20], were coupled to a CM5 sensor chip by using *N*-hydroxysuccinimide (NHS) and 1-ethyl-3-(3-dimethylaminopropyl) carbodiimide (EDC). Approximately 250 RU of purified secreted E2 (sE2) in HBS-EP buffer (10 mM HEPES pH 7.4, 150 mM NaCl, 3 mM EDTA, 0.005% v/v Surfactant P20.) (GE Healthcare, BIAcore BR-1001-88) were captured by CBH-4D onto the surface of the chip. Another flow cell without sE2 capture was set as reference. The purified HC-84.XX scFv at concentrations ranging 1000 nM-31.25 nM (with two-fold serial dilution) was injected for 2 minutes using a flow rate of 30 µl/min. Dissociation of bound HC-84.XX scFv in HBS-EP buffer flow was followed for 3 min. The surfaces (E2 and HC84.XX scFv) were regenerated after each cycle using regeneration solution (10 mM glycine-HCl, pH 2.5). All sensorgrams were double-referenced before data analysis. First, the response from the reference flow cell (without E2) was subtracted. Second, the response from an average of two-blank injections (0 nM E2) of HBS-EP buffer was subtracted. The sensorgrams (duplicates for each concentration) were globally fit with parameters *K*_on_ (association rate constant) and *K*_off_ (dissociation rate constant) using Scrubber 2.0 (Center for Biomolecular Interaction Analysis, University of Utah, UT). *K_D_* was calculated as *K*_off_/*K*_on_.

### Quantitative enzyme-linked immunoassays

ELISA were performed as described [68] to measure antibody binding to the wt E1E2 from different genotypes or mutant E2 glycoproteins and to measure E2 binding to CD81. Briefly, microtiter plates were prepared by coating each well with 500 ng of GNA and blocking with 2.5% non-fat dry milk and 2.5% normal goat serum. Lysates of cells expressing wt HCV E1E2 from different genotypes, mutant E1E2, denatured E1E2 proteins or pelleted HCVpp were captured by GNA onto the plate and later bound by a range of 0.01–100 µg/ml of HMAb. E1E2 protein was denatured by incubation with 0.5% sodium dodecyl sulfate and 5 mM dithiothreitol for 15 min at 56°C. The bound HMAb was detected by incubation with alkaline phosphatase-conjugated goat anti-human IgG (Promega; Madison, WI), followed by incubation with *p*-nitrophenyl phosphate for color development. Absorbance was measured at 405 nm and 570 nm. Data was analyzed for statistical significance by unpaired student's *t*-test, using Prism software (GraphPad Software).

In the case of the peptide (“epitope II" [23], [24]) binding assay, biotinylated peptide at 2 µg/ml was captured in microtiter plates by streptavidin. The wells were then incubated with either antibodies at 10 µg/ml or human sera at 1∶100 dilution. Binding was detected after anti-human IgG-horseradish peroxidase incubation and TMB peroxidase substrate color development. For the peptide competition assay, E2 protein expressed in 293 T cells was captured in microtiter plates by GNA. The wells were then incubated with antibodies that were pre-incubated with peptide at concentrations 0, 2, 5, 10, 20 and 40 µg/ml. Antibody binding was detected as described above.

### Western Blot

Partially purified HCVpp, obtained by pelleting virus through a 20% sucrose cushion, were dissolved in TNE buffer (10 mM Tris-HCL, pH 7.4, 150 mM NaCl and 1 mM EDTA). Equal amounts of HCVpp samples adjusted by the amount of HIV p24 incorporation were boiled for 3 min in Laemmli reducing sodium dodecyl sulfate (SDS) sample buffer and were electrophoresed in pre-cast 10% polyacrylamide gel (Invitrogen, Carlsbad, CA). Following SDS-PAGE, separated proteins were transferred to nitrocellulose membranes and were immunoblotted with anti-E2 HMAb, HC-33.1 or MAb 6/82a, and anti-E1 HMAb, H-111. HIV p24 was identified by an anti-HIV p24 antibody (Invitrogen) as a loading control. After washing, the blots were probed with secondary antibodies (horseradish peroxidase-conjugated anti-human or anti-mouse IgG from Santa Cruz Biotech) and visualized by enhanced chemilumminescence (Amersham Pharmacia). Western images were captured using ChemiDoc imager system (Bio-Rad, Richmond, CA).

### Inhibition of binding of E2 glycoprotein to CD81

HMAbs at different concentrations were incubated for 20 min at 4°C with lysates of cells expressing wt H77c E1E2. The mixture was added to microtiter plates pre-coated with anti-GST that captured recombinant fusion proteins containing the large extracellular loop of human CD81 fused to glutathione S-transferase. After 1 hr incubation at 4°C with gentle agitation, the wells were washed and 5 µg/ml of anti-*cmyc* was added to the wells, followed by incubation and addition of 100 µl/well of 1/10,000-diluted alkaline phosphatase-conjugated anti-mouse IgG (Promega, Madison, WI). After color development, the plate was read at 405/570 nm using spectroMax 190. The percentage of binding inhibition was calculated as reduction of E2 binding to CD81 compared to the value that was obtained in the absence of antibody. Background signal for binding of E2 to human CD81 was determined from wells coated with murine CD81-LEL. Signals obtained with biotinylated-CBH-4D and E2 in the presence of competing antibody were compared to signals obtained from biotinylated-CBH-4D and E2 in the absence of competing antibody.

### Epitope mapping

Epitope mapping was performed using alanine substitution mutants of three defined E2 regions (region 1: aa418–446; region 2: aa526–536; region 3: aa611–617) by ELISA. Alanine substitution mutants were constructed in plasmids carrying the 1a H77C E1E2 coding sequence (GenBank accession nos. AF009606) as previously described [43]. All the mutations were confirmed by DNA sequence analysis (Sequetech, Mountain View, CA) for the desired mutation and for exclusion of unexpected residue changes in the full-length E1E2 encoding sequence. The resulting plasmids were transfected into HEK293T cells for transient protein expression using the calcium-phosphate method. The mutated constructs were designated X#Y, where # is the residue location in H77C, X denotes the single-letter code for the H77C amino acid, and Y denotes the altered amino acid.

### Generation of mutant viruses escaping neutralization by HC-84 antibodies

JFH-1 2a HCVcc was employed in studies to isolate escape variants from HC-84 HMAbs and performed essentially as described [43], [56] and diagramed in Figure S3. Briefly, Huh7.5 cells (3.2×10^4^/ml) seeded 24 hrs previously in a 24-well plate were inoculated with a mixture of HCVcc (1×10^4^ FFU) and test antibody. The initial concentration of the neutralizing antibody employed to isolate escape HCVcc mutants was adjusted to the 50% inhibitory concentration (IC_50_) of the antibody against the 2a HCVcc. Infectious virus was first incubated with the selection antibody for 1 hr at 37°C prior to inoculation onto naïve Huh7.5 cells. This was followed by a second incubation for 3 hrs at 37°C before the medium was replaced with fresh medium containing the same antibody concentration. The cultures were maintained for three days in the presence of individual HC-84 antibody or R04 (as mock human IgG selection). The cells were collected for analysis by indirect immunofluorescent assay (IFA) and the extracellular virus was harvested for virus titration, the next passage of selection, and stored for future viral sequence analysis. The entire process constituted one passage of infectious virus under a specified antibody concentration. At each antibody concentration, the virus was repeatedly passaged until the virus titer reached 1×10^4^ FFU/ml. The number of passages required for this purpose varied from antibody to antibody. If the virus titer was ≥10^4^ FFU/ml, extracellular virus was subjected then to the next round of higher antibody concentration. Starting at IC_50_, the antibody concentration was progressively increased (0.002, 0.001, 0.005, 0.01, 0.05, 0.1, 0.5, 1, 5, 10 and 100 µg/ml). Viral growth was measured by FFU assay and the emergence of escape variants was monitored weekly by two-color confocal immunofluorescence microscopy staining with the respective neutralizing antibody and an anti-NS3 antibody. Confocal immunofluorescence microscopy, focus-forming unit (FFU) assay used in virus titers determination and viral yield assay were performed as previously described [43], [50].

Selected viral supernatants were used for virus amplification followed by sequencing E2 genes to map the escape mutations. Viral supernatants were used for neutralization studies against escape mutants and as a source of virus stock. If and when virus under antibody selection reached an undetectable level, the selection antibody was withdrawn from the medium, and the culture was continued and monitored for an additional two passages.

### Sequence analysis

Total RNA or viral RNA from virus-infected cells or virus-containing culture supernatant was extracted using commercial kits (Qiagen, Valenica, CA). cDNA of the E2 glycoprotein was synthesized with SuperScript III reverse transcriptase (Invitrogen,Carlsbad, CA) by using primer p7rev CCCGACCCCTGATGTGCCAAGC in a 20-µl reaction of the manufacturer's recommended buffer. Subsequent amplification was performed in a 50-µl reaction using the Expend High Fidelity PCR system (Roche Applied Sciences, Indianapolis, IN) and primers E1fwd GGTCATCATAGACATCGTTAGC and p7rev CCCGACCCCTGATGTGCCAAGC. The PCR consisted of 30 cycles at 94°C for 60 seconds, 45°C for 60 seconds, and 72°C for 90 seconds. A total of 2 µl of the resulting PCR product was used as template for a nested amplification, using primer pair E2F GGCACCACCACCGTTGGAGGC & E2R TGCTTCGGCCTGGCCCAACAAGAT. This second round of PCR comprised 25 cycles at 94°C for 60 seconds, 55°C for 60 seconds, and 72°C for 90 seconds. In some cases, when viral titer was low and failed to amplify the E2 gene, the number of PCR cycles in the nested round was increased. The PCR products were purified with the QIAquick gel extraction kit (Qiagen, Valencia, CA), ligated into the TOPO cloning vector (Invitrogen, Carlsbad, CA), and individual clones containing an insert of the expected size were sequenced in both sense and antisense strands (ElimBiopharm, Hayward, CA).

## Supporting Information

Figure S1 **Dose-dependent neutralization of JFH1-based genotype 3a (S52) recombinant virus as determined by FFU reduction with HMAb HC84.1.** Two batches of the same antibody were evaluated, as designated. Infectious virus inoculum was incubated with each HMAb at 0.005–50 µg/ml followed by inoculation onto Huh7.5 cells. Cells were immunostained with a MAb to NS5A antigen at 45 hrs p.i., and enumerated by FFU. The error bars are SEMs of 6 replicates compared with 6 replicates of virus only. The concentration required to reach 50% neutralization (IC_50_) is calculated by nonlinear regression.
(TIF)

Figure S2 **Measurement of HC-84 scFv affinity to HCV 1a E2 by SPR.** (**A–H**) Association and dissociation curves obtained for each HC-84 scFv against immobilized 1a H77C E2 captured by CBH-4D [18], [19], [20] as measured by BIAcore 3000. Each scFv was evaluated at concentrations ranging 1000 nM-31.25 nM (with two-fold serial dilutions).
(TIF)

Figure S3 **Isolation of mutant viruses escaping virus neutralization.** Huh7.5 cells were inoculated with a mixture of JFH1 2a HCVcc and test antibody, at an initial concentration that was adjusted to the 50% inhibitory concentration (IC_50_). After 3 hrs at 37°C, the medium was replaced with fresh medium containing the same antibody concentration. The cultures were maintained for three days in the presence of individual test antibody. The cells were collected for analysis by indirect immunofluorescent assay (IFA) and the extracellular virus was harvested for virus titration, the next passage of selection and stored for future viral sequence analysis. The entire process constituted one passage of infectious virus under a specified antibody concentration. At each antibody concentration, the virus was repeatedly passaged until the virus titer reached 1×10^4^ FFU/ml. The number of passages required for this purpose varied from antibody to antibody. If and when virus under antibody selection reached an undetectable level, the selection antibody was withdrawn from the medium, and the culture was continued and monitored for an additional two passages.
(TIF)
