# Supplementary figures and images for: Human Monoclonal Antibodies to a Novel Cluster of Conformational Epitopes on HCV E2 with Resistance to Neutralization Escape in a Genotype 2a Isolate
Source: PLoS Pathog. 2012 Apr 12;8(4):e1002653. doi: 10.1371/journal.ppat.1002653 (PMC3325216; doi:10.1371/journal.ppat.1002653)

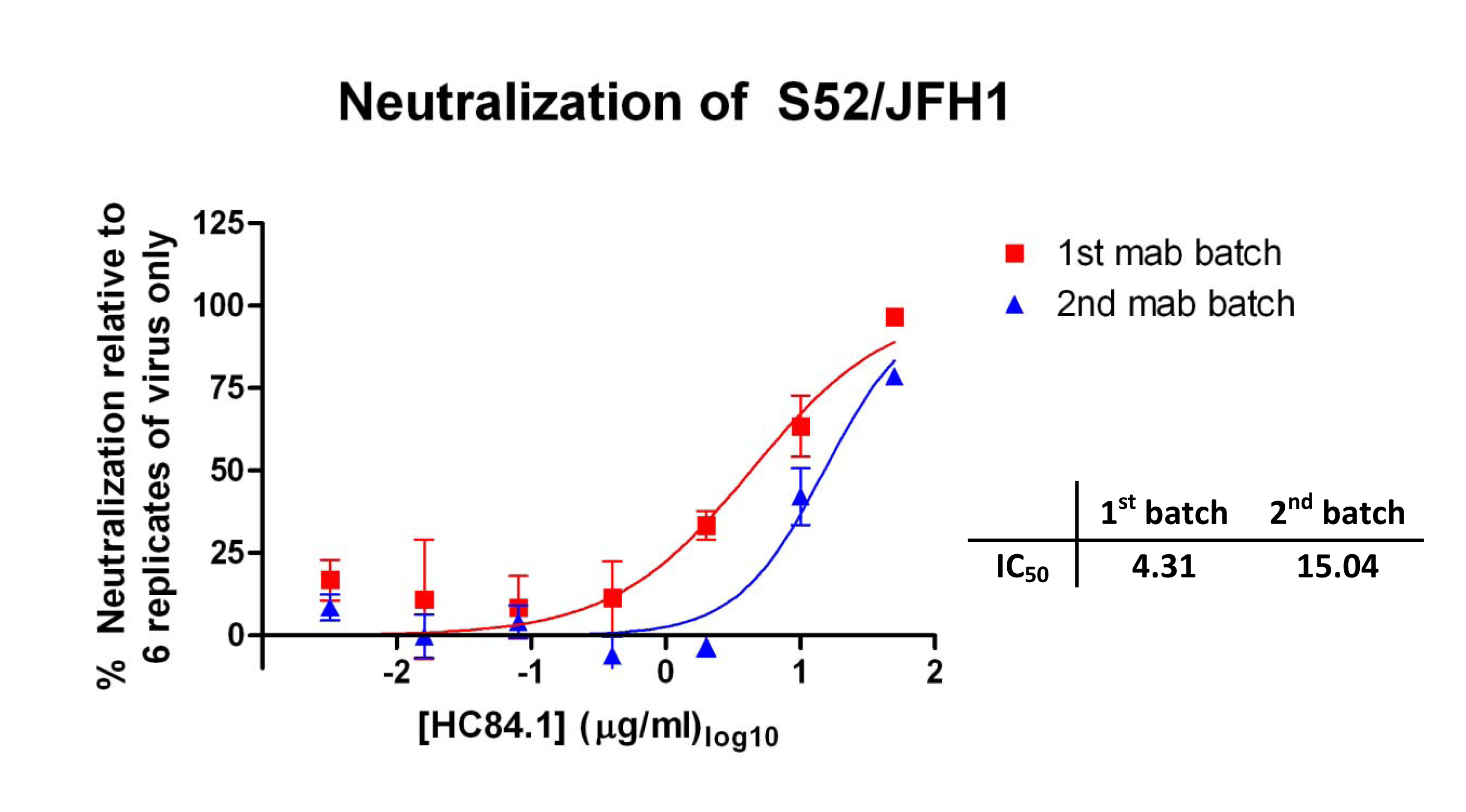

Supplement: Figure S1 — Dose-dependent neutralization of JFH1-based genotype 3a (S52) recombinant virus as determined by FFU reduction with HMAb HC84.1. Two batches of the same antibody were evaluated, as designated. Infectious virus inoculum was incubated with each HMAb at 0.005–50 µg/ml followed by inoculation onto Huh7.5 cells. Cells were immunostained with a MAb to NS5A antigen at 45 hrs p.i., and enumerated by FFU. The error bars are SEMs of 6 replicates compared with 6 replicates of virus only. The concentration required to reach 50% neutralization (IC50) is calculated by nonlinear regression. (TIF) [file ppat.1002653.s001.tif]

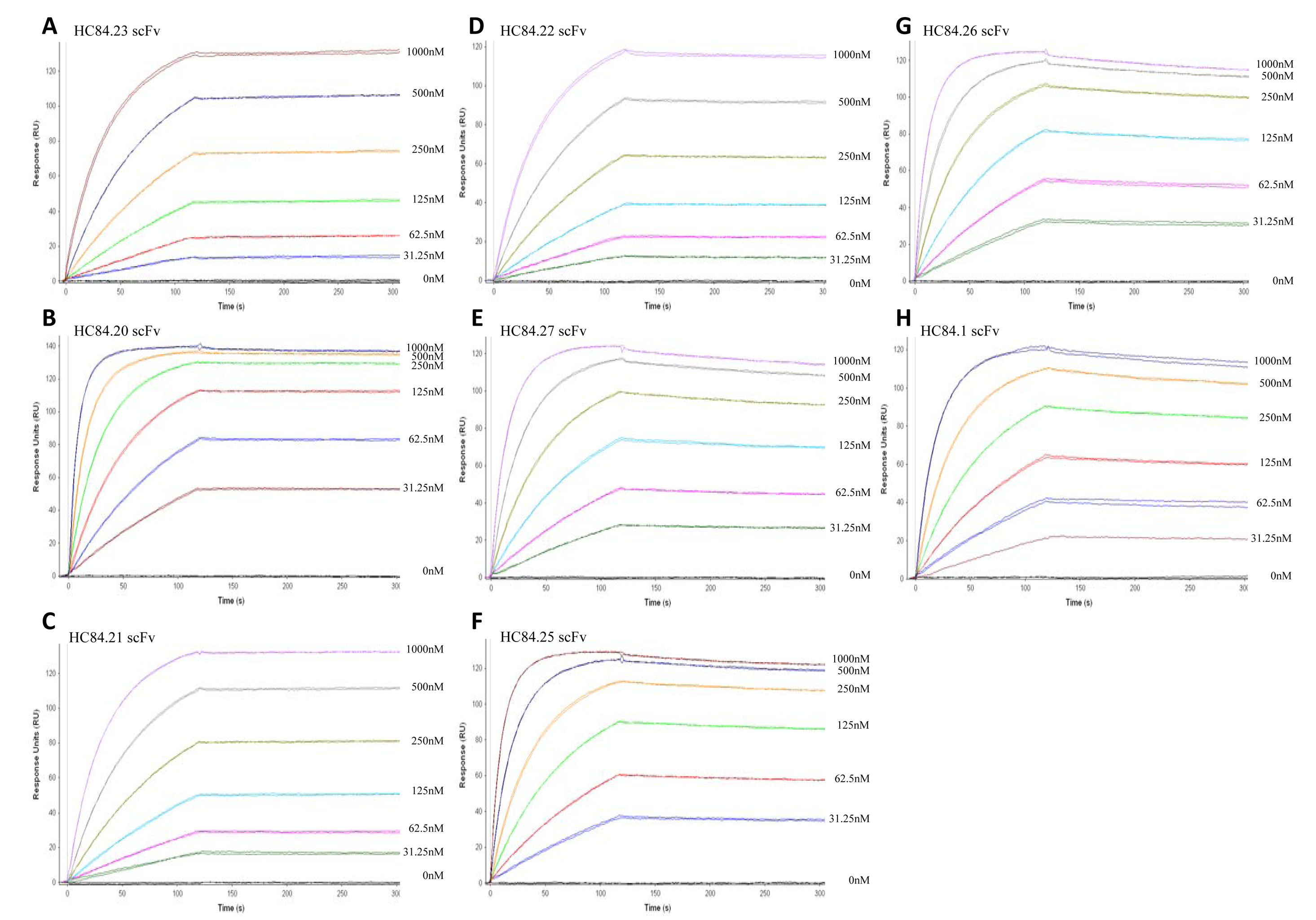

Supplement: Figure S2 — Measurement of HC-84 scFv affinity to HCV 1a E2 by SPR. (A–H) Association and dissociation curves obtained for each HC-84 scFv against immobilized 1a H77C E2 captured by CBH-4D [18], [19], [20] as measured by BIAcore 3000. Each scFv was evaluated at concentrations ranging 1000 nM-31.25 nM (with two-fold serial dilutions). (TIF) [file ppat.1002653.s002.tif]

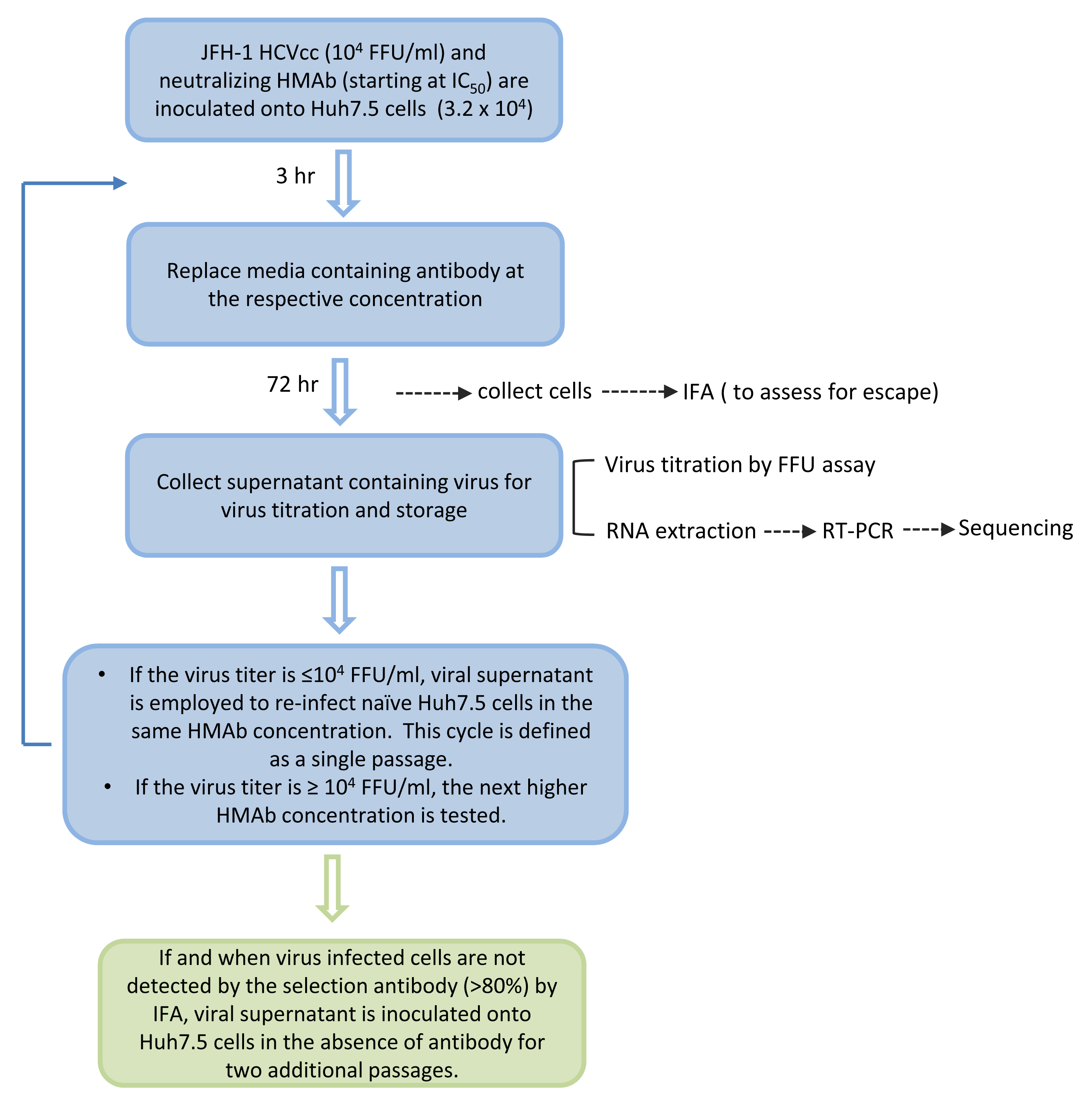

Supplement: Figure S3 — Isolation of mutant viruses escaping virus neutralization. Huh7.5 cells were inoculated with a mixture of JFH1 2a HCVcc and test antibody, at an initial concentration that was adjusted to the 50% inhibitory concentration (IC50). After 3 hrs at 37°C, the medium was replaced with fresh medium containing the same antibody concentration. The cultures were maintained for three days in the presence of individual test antibody. The cells were collected for analysis by indirect immunofluorescent assay (IFA) and the extracellular virus was harvested for virus titration, the next passage of selection and stored for future viral sequence analysis. The entire process constituted one passage of infectious virus under a specified antibody concentration. At each antibody concentration, the virus was repeatedly passaged until the virus titer reached 1×10^4^ FFU/ml. The number of passages required for this purpose varied from antibody to antibody. If and when virus under antibody selection reached an undetectable level, the selection antibody was withdrawn from the medium, and the culture was continued and monitored for an additional two passages. (TIF) [file ppat.1002653.s003.tif]
